# Improvement in quality of life after asfotase alfa treatment in adults with pediatric-onset hypophosphatasia: data from 5 patient-reported outcome measures

**DOI:** 10.1093/jbmrpl/ziae062

**Published:** 2024-05-07

**Authors:** Kathryn M Dahir, Steven W Ing, Chad Deal, Andrew Messali, Toby Bates, Eric T Rush

**Affiliations:** Endocrinology and Diabetes, Vanderbilt University Medical Center, Nashville, TN 37232, United States; Division of Endocrinology, Diabetes, and Metabolism, Wexner Medical Center, Ohio State University, Columbus, OH 43210, United States; Department of Rheumatology, Center for Osteoporosis and Metabolic Bone Disease, Cleveland Clinic, Cleveland, OH 44195, United States; Health Economics and Outcomes Research (AM) and Medical Affairs (CD), Alexion, AstraZeneca Rare Disease, Boston, MA 02210, United States; Health Economics and Outcomes Research (AM) and Medical Affairs (CD), Alexion, AstraZeneca Rare Disease, Boston, MA 02210, United States; Division of Clinical Genetics, Children’s Mercy Kansas City, Kansas City, MO 64108, United States; Department of Pediatrics, University of Missouri – Kansas City School of Medicine, Kansas City, MO 64108, United States

**Keywords:** hypophosphatasia, asfotase alfa, quality of life, real-world evidence, patient-reported outcome measures

## Abstract

Hypophosphatasia (HPP) is a rare, inherited metabolic disorder caused by deficient tissue-nonspecific alkaline phosphatase activity. This study assessed the impact of treatment with asfotase alfa on patient-reported outcomes (PROs) in adults with pediatric-onset HPP. A longitudinal, telephone-based survey was administered to eligible individuals enrolled in a patient support program. Interviews were conducted at study entry (prior to asfotase alfa initiation) and after 3, 6, and 12 mo. PROs—Patient Health Questionnaire-9 [PHQ-9], Work Productivity and Activity Impairment Questionnaire: Specific Health Problem [WPAI:SHP], Patient-Reported Outcomes Measurement Information System 29 [PROMIS-29], and Routine Assessment of Patient Index Data 3 [RAPID3]—were assessed at each time point. Appropriate statistical tests were performed to assess score changes. Among 50 enrolled patients (mean age: 46 yr [SD: 15.4]; 80% female; 94% White), 49 were evaluable at 3 mo, 44 at 6 mo, and 29 at 12 mo. By month 3, statistically significant improvements from baseline were detected in PHQ-9 scores (10.6 vs 5.8 [*P <* .0001]), PROMIS-29 domain scores (overall physical function: 38.0 vs 43.0 [*P =* .001]; anxiety: 57.5 vs 51.5 [*P =* .0011]; fatigue: 63.3 vs 55.3 [*P <* .0001]; sleep disturbances: 58.8 vs 54.3 [*P =* .0099]; ability to participate in social roles and activities: 42.6 vs 47.7 [*P =* .0012]; and pain interference: 63.8 vs 58.4 [*P =* .001]), and RAPID3 domain scores (functional status: 2.7 vs 1.1 [*P <* .0001]; pain tolerance: 6.0 vs 3.2 [*P <* .0001]; and global health estimate: 5.1 vs 2.7 [*P <* .0001]). Improvements persisted at month 12. Patients also showed improvements in WPAI:SHP domain scores at month 6 (presenteeism: 39.6% vs 14.1% [*P <* .0001] and work productivity loss: 41.9% vs 14.1% [*P <* .0001]). Treatment with asfotase alfa was associated with improved quality of life across several domains.

## Graphical Abstract

**Graphical Abstract:**
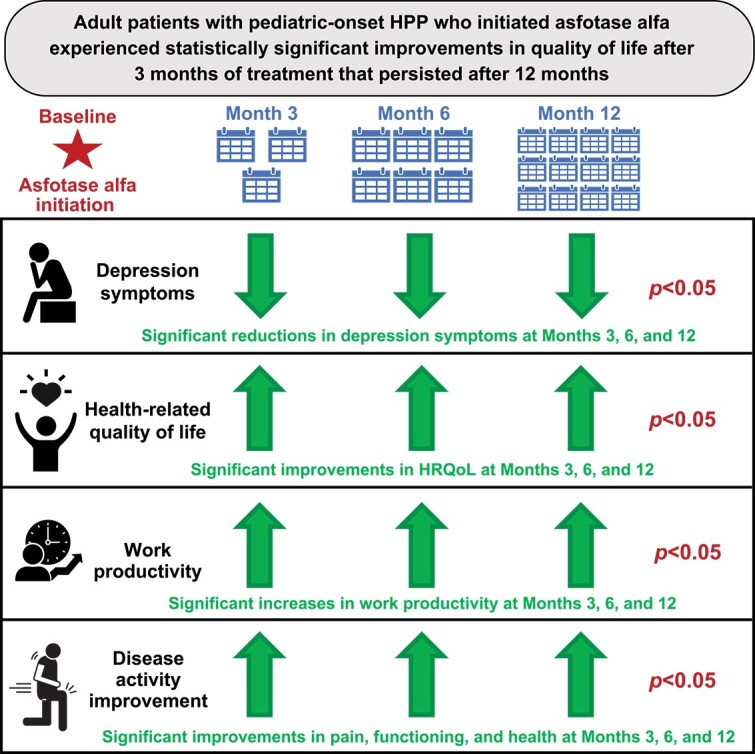

## Introduction

Hypophosphatasia (HPP) is a rare, inherited metabolic disorder caused by deficient activity of the tissue-nonspecific isoenzyme of alkaline phosphatase.^1^ This deficiency results in impaired bone mineralization, leading to numerous skeletal, dental, and other systemic problems.^1^ There is substantial heterogeneity in the clinical presentation of HPP, including manifestations such as joint pain, impaired mobility, fractures, and muscle weakness.^2^ These manifestations can substantially limit activities of daily living and negatively impact quality of life (QoL).^3^^,^^4^ In Seefried et al., more than half of patients reported that HPP negatively affected both their physical and mental functioning.^4^ Many patients also require mobility devices and physical therapy to support their activities of daily living.^3^

The human recombinant enzyme replacement therapy asfotase alfa (Strensiq^®^, Alexion, AstraZeneca Rare Disease) is currently the only US Food and Drug Administration-approved treatment for patients with perinatal-onset (symptom onset in utero or at birth), infantile-onset (at age < 6 mo), and juvenile-onset (at age ≥ 6 mo to <18 yr) HPP.^5^ Data from clinical trials support the long-term efficacy and safety of asfotase alfa^6-8^; however, limited data on the use of asfotase alfa in routine clinical practice are available. One real-world cohort study found that asfotase alfa significantly improved physical functioning and health-related QoL (HRQoL) among 22 adult patients with pediatric-onset HPP and this improvement was sustained over 24 mo.^9^^,^^10^ A 2023 analysis of data from the Global HPP Registry also found that adults with HPP who received asfotase alfa experienced improvements in mobility, physical function, and pain.^11^ However, more research is needed to better understand treatment response and establish appropriate metrics for evaluation.

This prospective, observational study assessed the real-world impact of asfotase alfa treatment on HRQoL among adult patients with pediatric-onset HPP using standardized patient-reported outcome measures (PROMs). PROMs are used to evaluate the effectiveness of various treatments from the patient perspective and are relied upon by health care stakeholders when performing health technology assessments.^12^ Although meaningful endpoints for infants and children with HPP may be measured with survival time, respiratory status, and evidence of rickets, quantifying disease activity and treatment efficacy in adult patients with HPP is more challenging. Recent published guidance suggests that pain and QoL scales be used to monitor disease improvement among adults with HPP treated with asfotase alfa.^5^ Currently, the Global HPP Registry uses the Short Form-36 version 2 and Health Assessment Questionnaire to assess patient QoL over time. Our study builds on the growing body of evidence supporting the real-world effectiveness of asfotase alfa by using additional measures of QoL, mental health, and work productivity loss among patients with HPP.

## Materials and methods

### Study design and population

This was a prospective, observational, longitudinal, noninterventional study of patients in the USA aged 18 or older who were diagnosed with pediatric-onset HPP and were newly initiating asfotase alfa. Patients were already enrolled in a patient support program (OneSource) and were eligible for the study if they intended to initiate asfotase alfa treatment. To remain in the study, patients had to begin treatment within 8 mo of study enrollment. Patients were interviewed at study entry and after 3, 6, and 12 mo of asfotase alfa treatment. There were no study-mandated visits or treatments; all patients were undergoing routine clinical care.

### Patient-reported outcome measures

Data were collected from patients using 5 PROMs. The Patient Health Questionnaire-9 (PHQ-9) is a 9-question survey used to evaluate the presence and severity of depression in the general population.^13^ The Work Productivity and Activity Impairment Questionnaire: Specific Health Problem (WPAI:SHP) is a 6-question survey that assesses absenteeism, presenteeism, and daily activity impairment attributable to a specific health problem.^14^ The Patient-Reported Outcomes Measurement Information System 29 (PROMIS-29) global form is a 29-question instrument that assesses overall physical function, anxiety, depression, fatigue, sleep disturbance, social roles and activities, and pain interference; scores in each domain are normalized to a general adult population mean of 50 and standard deviation (SD) of 10.^15^ The Routine Assessment of Patient Index Data 3 (RAPID3) is a disease activity index originally developed for use in rheumatoid arthritis but was used in this study because of the functional impairments and mobility challenges seen in patients with HPP.^16^ Finally, the 14-item Treatment Satisfaction with Medications Questionnaire (TSQM) was used to assess patients’ satisfaction with their asfotase alfa treatment at the end of the study period.^17^ A summary of the PROMs used in this study is shown in Table 1.

**Table 1:** Overview of patient-reported outcome measures used in this study.

	**Patient Health Questionnaire** **(PHQ-9)**	**Work Productivity and Activity Impairment Questionnaire: Specific Health Problem (WPAI:SHP)**	**Patient-Reported Outcomes Measurement Information System (PROMIS-29)**	**Routine Assessment of Patient Index Data (RAPID3)**	**Treatment Satisfaction with Medications Questionnaire** **(TSQM)**
**Domain(s)**	Depression	Work productivity (presenteeism, absenteeism) Daily activities	Physical functioning Anxiety Depression Fatigue Sleep disturbances Social roles and activities Pain interference	Physical function Pain Patient global assessment of disease activity	Side effects Effectiveness Convenience Global Satisfaction
**Recall period**	Past 2 wk	Past 7 d	Past 7 d (except for physical functioning, which has no specified time frame)	Past week (except for patient global estimate, which is measured “at this time”)	Past 7 d
**Scoring**	Range: 0–27 Severity categories: minimal (0–4), mild (5–9), moderate (10–14), moderately severe (15–19), severe (20–27)	Scores are multiplied by 100 to be expressed as impairment percentages for each domain	Standardized *t*-scores are computed, with a general adult population mean of 50 and standard deviation of 10 for each domain	Range: 0-10; Cumulative scores are converted to weighted scores using a published conversion table	Range: 0–100
**Score directionality**	Higher scores indicate more severe depression	Higher percentages indicate greater impairment	Higher scores on physical function and social roles and activities domain indicate better functioning; higher scores on all other domains indicate worse HRQoL	Higher scores indicate more active disease	Higher scores indicate higher overall treatment satisfaction
**Assessment timing**	Baseline; months 3, 6, and 12 post-initiation	Baseline; months 3, 6, and 12 post-initiation	Baseline; months 3, 6, and 12 post-initiation	Baseline; months 3, 6, and 12 post-initiation	Month 12 post-initiation

Abbreviation: HRQoL, health-related quality of life.

### Data collection

Baseline interviews were conducted on the day the patient enrolled in the study. Newly enrolled patients who had not initiated asfotase alfa within 4 mo of completing the baseline interview were asked to repeat the baseline interview at month 4 and were given an additional 4 mo to begin treatment.

After asfotase alfa initiation, follow-up interviews were conducted at months 3, 6, and 12 of treatment. At each of these time points, patients completed all the PROMs, except the TSQM, which was completed only after 12 mo of treatment. Patients also reported any concomitant medication use, treatment compliance, and treatment discontinuation. Patients who reported asfotase alfa discontinuation at any time point were evaluated at that time point, queried about their reason(s) for discontinuing asfotase alfa, and then subsequently removed from the study. All interviews were conducted over the telephone.

### Primary outcome measure

The primary outcome measure of this study was change in HRQoL during asfotase alfa treatment, as measured by changes from baseline to months 3, 6, and 12 in scores on the PHQ-9, WPAI:SHP, PROMIS-29, and RAPID3, and at month 12 on the TSQM.

### Statistical analysis

Data were summarized descriptively, as counts and percentages for categorical variables and means and SDs for continuous variables. All PROMs were scored based on published scoring documentation.^13-17^ Score changes were analyzed using McNemar’s test or Cochran–Mantel–Haenszel test for paired proportions (for categorical variables) and paired *t*-tests (for continuous variables), as appropriate. Only patients who had data for both time points being compared were included in the analyses. For all tests, results were considered significant at *P* < .05. Further, owing to the descriptive nature of the analysis, no adjustments were made for multiple comparisons.

## Results

### Study population

A total of 50 adults with a diagnosis of pediatric-onset HPP were included in the analysis. Patient demographics at baseline are presented in Table 2. The mean (SD) age of the study population was 46.0 (15.4) yr; 80% of patients were female, and 94% were White.

**Table 2:** Patient demographics at baseline (*n* = 50).

Characteristic	*n*	%
Age (mean, SD)	46	15.4
Age group		
18–40	21	42.0
41–60	18	36.0
61+	11	22.0
Gender		
Female	40	80.0
Male	10	20.0
Race		
White	47	94.0
Non-White	3	6.0
Ethnicity		
Hispanic or Latino/a	0	0
Not Hispanic or Latino/a	48	96.0
Other/Unknown	2	4.0

### Patient-reported outcome measures

#### Depression

**Figure 1:**
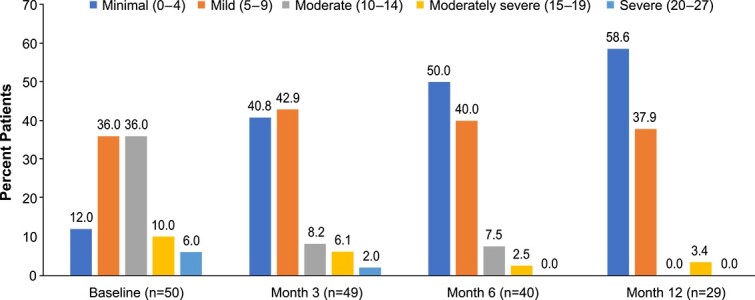
Percentage of patients in PHQ-9 depression severity categories at baseline and months 3, 6, and 12. Abbreviation: PHQ-9, Patient Health Questionnaire-9.

A total of 50, 49, 40, and 29 patients completed the PHQ-9 at baseline and months 3, 6, and 12, respectively. The percentage of patients with moderately severe depression decreased from 10.0% to 3.4% from baseline to month 12, and the percentage of patients with severe depression decreased from 6.0% to 0.0% over the 12 months (Figure 1). Mean (SD) PHQ-9 scores decreased (improved) significantly from baseline to month 3 (10.6 [5.1] vs 5.8 [4.9]; *P* < .0001) and continued to decrease at months 6 and 12. Full results of the PHQ-9 are presented in Table S1.

#### Work productivity and activity impairment

A total of 50, 49, 40, and 29 patients completed the WPAI:SHP at baseline and months 3, 6, and 12, respectively, though only patients who were employed (*n* = 28 at baseline, 28 at month 3, 22 at month 6, and 12 at month 12) were included in the analysis. By month 12, percent impairment while working (presenteeism) decreased from baseline from 39.6% to 14.0% (*P* < .0001), percent overall work productivity loss decreased from 41.9% to 20.0% (*P* < .0001), and percent daily activity impairment decreased from 64.0% to 30.0% (*P* < .0001).

**Figure 2:**
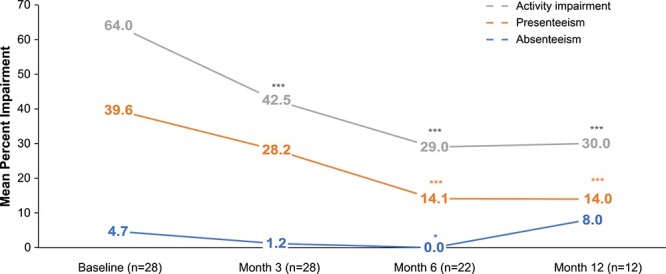
Percent impairment results across WPAI:SHP domains at baseline and months 3, 6, and 12. Results of the WPAI:SHP are reported only for those who responded “yes” to “Are you currently employed (working for pay)?”. ^*^Significant change from baseline at the *P* < .05 level. ^*^^*^^*^Significant change from baseline at the *P* < .0001 level. Abbreviation: WPAI: SHP, Work Productivity and Activity Impairment Questionnaire: Specific Health Problem.

Percent work time missed (absenteeism) also significantly decreased from baseline to month 6 (4.7% to 0.0% [*P* = .025]); however, absenteeism increased again at month 12. Full results of the WPAI:SHP are presented in Figure 2 and Table S2.

#### Health-related QoL

PROMIS-29 scores showed improvements in patients’ HRQoL throughout the study. A total of 50, 49, 40, and 29 patients completed the PROMIS-29 at baseline and months 3, 6, and 12, respectively. Improvements in mean (SD) scores were significant from baseline to month 3 and remained significant at month 12 across multiple domains, including overall physical function (38.0 [5.8] at baseline vs 46.5 [8.7] at month 12; *P <* .0001), anxiety (57.5 [8.9] vs 47.9 [7.3]; *P <* .0001), fatigue (63.3 [8.0] vs 51.3 [8.5]; *P <* .0001), sleep disturbances (58.8 [8.1] vs 51.6 [8.9]; *P =* .0008), ability to participate in social roles and activities (42.6 [6.9] vs 50.7 [8.9]; *P =* .0001), and pain interference (63.8 [6.8] vs 54.9 [9.1]; *P <* .0001). Reductions in mean depression scores were significant at month 6 and month 12 (52.6 [8.4] vs 47.4 [6.7]; *P =* .003). Full PROMIS-29 results can be found in Table 3 and Table S3.

**Table 3:** PROMIS-29 results.

**Domain**	**Mean (SD) score**		
	**Month 3 (vs Baseline)**	**Month 6 (vs Baseline)**	**Month 12 (vs Baseline)**
**Physical functioning**	43.0 (8.4) vs 38.0 (5.8), *P* = .001	44.6 (8.3) vs 38.0 (5.8), *P* < .0001	46.5 (8.7) vs 38.0 (5.8), *P* < 0.0001
**Anxiety**	51.5 (8.7) vs 57.5 (8.9), *P* = .001	49.4 (8.0) vs 57.5 (8.9), *P* < .0001	47.9 (7.3) vs 57.5 (8.9), *P* < .0001
**Depression**	49.8 (8.4) vs 52.6 (8.4), *P* = .090	46.6 (7.4) vs 52.6 (8.4), *P* = .0005	47.4 (6.7) vs 52.6 (8.4), *P* = .003
**Fatigue**	55.3 (9.9) vs 63.3 (8.0), *P* < .0001	51.9 (8.8) vs 63.3 (8.0), *P* < .0001	51.3 (8.5) vs 63.3 (8.0), *P* < .0001
**Sleep disturbance**	54.3 (8.9) vs 58.8 (8.1), *P* = .001	52.3 (7.5) vs 58.8 (8.1), *P* = .0002	51.6 (8.9) vs 58.8 (8.1), *P* = .0008
**Social roles and activities**	47.7 (8.3) vs 42.6 (6.9), *P* = .001	50.4 (8.3) vs 42.6 (6.9), *P* < .0001	50.7 (8.9) vs 42.6 (6.9), *P* = .0001
**Pain interference**	58.4 (8.7) vs 63.8 (6.8), *P* = .001	56.7 (7.1) vs 63.8 (6.8), *P* < .0001	54.9 (9.1) vs 63.8 (6.8), *P* < .0001

Abbreviation: PROMIS-29, Patient-Reported Outcomes Measurement Information System 29.

#### Disease activity

A total of 50, 49, 40, and 29 patients completed the RAPID3 at baseline and months 3, 6, and 12, respectively, and demonstrated improvement in functional status, pain tolerance, and global health assessment. Reductions in mean (SD) weighted total scores were significant at month 3 and remained significant at month 12 (4.6 [1.6] at baseline vs 2.4 [1.6] at month 12; *P* < .0001). Score reductions across RAPID3 domains (functional status, pain tolerance, and global health estimate) were also statistically significant at month 3 and remained significant at month 12.

**Figure 3:**
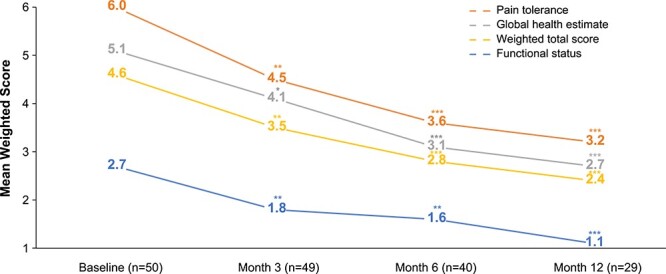
RAPID3 results at baseline and months 3, 6, and 12. Note: Functional status is based on questions 1a–1j of the RAPID3; pain tolerance refers to question 2 of the RAPID3; global health estimate refers to question 3 of the RAPID3. ^*^Significant change from baseline at the *P* < .05 level. ^*^^*^Significant change from baseline at the *P* < .01 level. ^*^^*^^*^Significant change from baseline at the *P* < .0001 level. Abbreviation: RAPID3, Routine Assessment of Patient Index Data 3.

Patients categorized as “near remission” or “low disease severity” increased from 6.0% at baseline to 24.4%, 42.5%, and 44.8% at months 3, 6, and 12, respectively. However, these increases were not assessed for statistical significance. Full RAPID3 results can be found in Figure 3 and Table S4.

#### Treatment satisfaction

Twenty-nine patients completed the TSQM after 12 months of treatment with asfotase alfa. Nearly three quarters (72.4%) of patients were satisfied, very satisfied, or extremely satisfied with treatment. Additionally, 93.1% of patients were somewhat, very, or extremely confident that taking asfotase alfa was a good thing for them. Although 44.8% of patients indicated experiencing some adverse effects, 69.2% of these patients rated the adverse effects as not at all or a little bothersome. Full results of the TSQM are available in Table S5.

### Concomitant medications

Use of concomitant medications remained largely the same or decreased slightly throughout the study. However, patient-reported use of analgesics (including narcotics) decreased from 20.0% at baseline to 10.3% at month 12. However, these findings were not analyzed for statistical significance. Data on patient use of concomitant medications are presented in Table S6.

### Treatment discontinuation

After 3 mo of treatment, 2 of 50 patients reported discontinuing asfotase alfa. At mo 6, 48 patients were contacted and 4 had discontinued, and at month 12, 37 patients were contacted and an additional 4 had discontinued. Reasons cited for treatment discontinuation included adverse effects and physician recommendation. Full results on treatment discontinuation are available in Table S7.

## Discussion

This study of a real-world cohort of adults with pediatric-onset HPP suggests that asfotase alfa is effective in improving physical functioning, fatigue, sleep, pain, work productivity, and emotional well-being. Statistically significant improvements across HRQoL domains were generally apparent by 6 mo of treatment. Findings also highlight the potential utility of PROMs validated in other disease areas in assessing treatment response in patients with HPP.

Baseline data across the PROMs used in this study demonstrate the high degree of disease burden in patients with HPP. Previous research has also shown that patients living with HPP often have diminished functional capacity and HRQoL resulting from systemic skeletal and muscular abnormalities, and many require assistive devices to support mobility.^3^^,^^4^ Our data show statistically significant improvements in PROMIS-29 and RAPID3 scores on the physical functioning domains after 12 mo of treatment with asfotase alfa. In a study by Genest et al., asfotase alfa was associated with significantly improved performance on mobility assessments, such as the Six-Minute Walk Test and the Timed Up & Go test.^9^ Additionally, a previous survey found that nearly two-thirds of adults with HPP had ever required assistive devices (eg, wheelchairs, crutches).^3^

The negative relationship between pain and HRQoL is well established,^18^ and patients living with HPP often experience significant bone and muscle pain.^3^^,^^9^ RAPID3 and PROMIS-29 score changes in the current study showed that patients treated with asfotase alfa had significant improvements in pain tolerance and interference. These findings are consistent with those of previous work. Clinical trial data reported by Kishnani et al.^6^ showed that patients experienced significant reductions in pain severity after 5 yr of treatment with asfotase alfa. Using registry data, Genest et al. found reductions in pain intensity among HPP patients treated with asfotase alfa; however, although pain intensity decreased after 6 mo of treatment, it increased again by month 12.^9^ Further research is needed to contextualize and support these findings, as different PROMs assess pain in different ways and patients’ perceptions of pain may change over time.

Results of the current study also suggest that treatment with asfotase alfa is associated with improved mental health. Patients with HPP have been found to score lower on measures of mental health than the general adult population.^3^ In this study, scores on the PHQ-9 decreased by more than 2-fold over 12 mo of treatment, and the percentage of patients experiencing mild depression (compared with moderate or severe depression) improved 5-fold, from 12% at baseline to 59% after 12 mo of treatment. These results were bolstered by findings from the PROMIS-29, where scores on anxiety and depression domains showed statistically significant improvements from baseline by months 3 and 6 of treatment, respectively, that were maintained through month 12.

This study is the first to quantitatively assess work productivity among adults with HPP, though evidence from patients with other diseases, such as rheumatoid arthritis, has shown that diminished functional ability is associated with work productivity impairment.^19^ As measured by the WPAI:SHP, work productivity and activity impairment were significantly improved in patients treated with asfotase alfa in this study. After 6 mo of treatment, presenteeism decreased from 39.6% to 14.1%, percent overall work productivity loss decreased from 41.9% to 14.1%, and percent daily activity impairment decreased from 64.0% to 29.0%. Work time missed (absenteeism) was generally low at all study time points, although it unexpectedly increased between months 6 and 12. However, just 12 patients were evaluable on the WPAI:SHP at month 12, and the observed increase may have been due to a single patient who reported 0% absenteeism at month 6 and 100% absenteeism at month 12. Given the small sample size in this study, future research should further explore the effects of asfotase alfa treatment on work productivity and activities of daily living of patients with HPP. Measures of work productivity loss (or gain) are often incorporated into cost-effectiveness analyses to inform health care decision-making, underscoring the importance of quantifying this aspect of disease burden.^20^

Our research highlights the utility of using existing PROMs to assess the burden of HPP and evaluate treatment effectiveness. Still, the use of PROMs in rare diseases poses some challenges.^12^ The limited number of patients makes it more difficult to validate existing PROMs and develop disease-specific instruments. The interpretation of PRO data also requires careful consideration. Although score changes over time may be statistically significant, evidence must be generated to establish data thresholds that correspond to clinically meaningful changes in patient well-being.^12^ Still, our data show improvements in HRQoL that correspond to minimal clinically important differences (MCIDs) in other disease areas. For example, previous research has found MCIDs in multiple PROMIS-29 domains of 3.6 to 4.6 points for patients with upper extremity fractures and 3.5 to 5.5 for patients with back pain.^21^^,^^22^ Our data show a change of at least 5 points from baseline to month 12 of asfotase alfa treatment across all domains. Similarly, some evidence suggests that a 6-point reduction in PHQ-9 score among patients with treatment-resistant depression represents a clinically substantial improvement in symptoms.^23^ In our study, the mean PHQ-9 score dropped by 6.4 points from baseline to month 12. Further, a RAPID3 cumulative score improvement of 3.8 points has been identified as clinically meaningful among patients with rheumatoid arthritis^24^; patients with HPP in our study experienced a cumulative score improvement of 5.5 points after 6 mo (mean [SD]: 13.8 [4.6] at baseline vs 8.3 [4.8] at month 6). However, it should be noted that the RAPID3 was specifically developed for use in rheumatoid arthritis and has not been used in HPP. Given the heterogeneity of HPP phenotypes, caution must be exercised in interpreting RAPID3 results in the current population. Other potential instruments should be evaluated for use in HPP.

This study has some limitations that merit discussion. Detailed clinical information, such as diagnostic testing results and severity of disease, were not collected by the patient support program; thus, any impact of such characteristics on PROM responses could not be explored. Further, as all information collected in this study was self-reported, it is not known what criteria may have been used by treating physicians to evaluate patients for asfotase alfa treatment. In addition, self-reported information is inherently subject to recall bias. There is also potential for a placebo response; for example, in patients with osteoarthritis, evidence has shown greater placebo effects with subjective measures than with objective functional measures, though the magnitude and duration of these placebo effects over time were not described.^25^ Notably, the positive effects from asfotase alfa were observed in subjective and objective measures over 12 mo, lessening these concerns. Future research could evaluate this phenomenon in patients with HPP treated with asfotase alfa using both patient-reported and objective assessments, ideally in a randomized trial setting.

This study may also be subject to attrition bias, as just 58% of patients enrolled at baseline remained in the study after 12 mo. The treatment status of patients lost to follow-up remains unclear; patients may have discontinued treatment, ceased to engage with the patient support program, or been lost for another reason. However, statistically significant improvements in most outcome measures were detected by months 3 and 6 of treatment, at which time points 98% and 88% of patients were evaluable, respectively. It should also be noted that no PROM has yet been validated in the HPP patient population. Further research and validation are needed to elucidate how statistical changes in scores on PROMs may correspond to clinically meaningful improvements. This study also used data from a limited number of patients in a single patient support program in the US, of whom most were White, employed, and female; this may restrict the generalizability of the results to the broader population adults with HPP. However, this investigation represents the largest assessment of adults with HPP treated with asfotase alfa in the real-world setting. Findings contribute to the growing body of evidence of the effectiveness of asfotase alfa and further establish the potential utility of commonly used HRQoL measures to assess burden of disease and treatment effects.

## Conclusion

These data from a real-world sample show that asfotase alfa is effective in improving HRQoL through improvements in physical functioning, pain, and mental health in adults with pediatric-onset HPP. Further, this study highlights the suitability of commonly used PROMs to evaluate the effectiveness of treatment and monitor improvements in patient HRQoL over time.

## Supplementary Material

Supplementary_Tables_RESUBMIT_ziae062

## Data Availability

Alexion, AstraZeneca Rare Disease will consider requests for disclosure of clinical study participant-level data provided that participant privacy is assured through methods like data de-identification, pseudonymization, or anonymization (as required by applicable law), and if such disclosure was included in the relevant study informed consent form or similar documentation. Qualified academic investigators may request participant-level clinical data and supporting documents (statistical analysis plan and protocol) pertaining to Alexion-sponsored studies. Further details regarding data availability and instructions for requesting information are available in the Alexion Clinical Trials Disclosure and Transparency Policy at https://alexion.com/our-research/research-and-development. Link to Data Request Form: https://alexion.com/contact-alexion/medical-information.
