# Supplementary material for: Improvement in quality of life after asfotase alfa treatment in adults with pediatric-onset hypophosphatasia: data from 5 patient-reported outcome measures
Source: JBMR Plus. 2024 May 7;8(8):ziae062. doi: 10.1093/jbmrpl/ziae062 (PMC11245646; doi:10.1093/jbmrpl/ziae062)
Supplement: Supplementary_Tables_RESUBMIT_ziae062 [file supplementary_tables_resubmit_ziae062.docx]

**Improvement in Quality of Life After Asfotase Alfa Treatment in Adults with Pediatric-onset Hypophosphatasia: Data from 5 Patient-reported Outcome Measures**

Kathryn M. Dahir and Steven W. Ing, et al.

# SUPPLEMENTAL TABLES

**Contents**

**Supplemental Table 1.** PHQ-9 Full Results

**Supplemental Table 2.** WPAI:SHP Full Results

**Supplemental Table 3.** PROMIS-29 Full Results

**Supplemental Table 4.** RAPID3 Full Results

**Supplemental Table 5.** TSQM Full Results

**Supplemental Table 6.** Concomitant Medication Usage

**Supplemental Table 7.** Asfotase alfa discontinuation

**Supplemental Table 1. PHQ-9 Full Results**

| \| **Item Responses** \| \| **Baseline (n = 50)** \| \| **Month 3 (n = 49)** \| \| **Month 6 (n = 40)** \| \| **Month 12 (n = 29)** \| \| \| --- \| --- \| --- \| --- \| --- \| --- \| --- \| --- \| --- \| --- \| \| **n** \| **%** \| **n** \| **%** \| **n** \| **%** \| **n** \| **%** \| \| **Little interest or pleasure in doing things** \| Not at all \| 8 \| 16.0 \| 30 \| 61.2 \| 25 \| 62.5 \| 22 \| 75.9 \| \| Several days \| 25 \| 50.0 \| 11 \| 22.4 \| 11 \| 27.5 \| 5 \| 17.2 \| \| More than half the days \| 10 \| 20.0 \| 3 \| 6.1 \| 3 \| 7.5 \| 1 \| 3.4 \| \| Nearly every day \| 7 \| 14.0 \| 5 \| 10.2 \| 1 \| 2.5 \| 1 \| 3.4 \| \| **Feeling down, depressed, or hopeless** \| Not at all \| 17 \| 34.0 \| 29 \| 59.2 \| 27 \| 67.5 \| 21 \| 72.4 \| \| Several days \| 22 \| 44.0 \| 16 \| 32.7 \| 10 \| 25.0 \| 8 \| 27.6 \| \| More than half the days \| 8 \| 16.0 \| 1 \| 2.0 \| 3 \| 7.5 \| 0 \| 0.0 \| \| Nearly every day \| 3 \| 6.0 \| 3 \| 6.1 \| 0 \| 0.0 \| 0 \| 0.0 \| \| **Trouble falling or staying asleep, or sleeping too much** \| Not at all \| 6 \| 12.0 \| 12 \| 24.5 \| 14 \| 35.0 \| 7 \| 24.1 \| \| Several days \| 11 \| 22.0 \| 17 \| 34.7 \| 13 \| 32.5 \| 15 \| 51.7 \| \| More than half the days \| 12 \| 24.0 \| 10 \| 20.4 \| 8 \| 20.0 \| 7 \| 24.1 \| \| Nearly every day \| 21 \| 42.0 \| 10 \| 20.4 \| 5 \| 12.5 \| 0 \| 0.0 \| \| **Feeling tired or having little energy** \| Not at all \| 2 \| 4.0 \| 7 \| 14.3 \| 9 \| 22.5 \| 7 \| 24.1 \| \| Several days \| 8 \| 16.0 \| 26 \| 53.1 \| 21 \| 52.5 \| 15 \| 51.7 \| \| More than half the days \| 14 \| 28.0 \| 10 \| 20.4 \| 5 \| 12.5 \| 4 \| 13.8 \| \| Nearly every day \| 26 \| 52.0 \| 6 \| 12.2 \| 5 \| 12.5 \| 3 \| 10.3 \| \| **Poor appetite or overeating** \| Not at all \| 23 \| 46.0 \| 31 \| 63.3 \| 29 \| 72.5 \| 19 \| 65.5 \| \| Several days \| 19 \| 38.0 \| 16 \| 32.7 \| 9 \| 22.5 \| 7 \| 24.1 \| \| More than half the days \| 5 \| 10.0 \| 2 \| 4.1 \| 2 \| 5.0 \| 2 \| 6.9 \| \| Nearly every day \| 3 \| 6.0 \| 0 \| 0.0 \| 0 \| 0.0 \| 1 \| 3.4 \| \| **Feeling bad about yourself—or that you are a failure or have let yourself or your family down** \| Not at all \| 24 \| 48.0 \| 34 \| 69.4 \| 31 \| 77.5 \| 21 \| 72.4 \| \| Several days \| 17 \| 34.0 \| 12 \| 24.5 \| 9 \| 22.5 \| 7 \| 24.1 \| \| More than half the days \| 4 \| 8.0 \| 1 \| 2.0 \| 0 \| 0.0 \| 1 \| 3.4 \| \| Nearly every day \| 5 \| 10.0 \| 2 \| 4.1 \| 0 \| 0.0 \| 0 \| 0.0 \| \| **Trouble concentrating on things, such as reading the newspaper or watching television** \| Not at all \| 14 \| 28.0 \| 28 \| 57.1 \| 24 \| 60.0 \| 19 \| 65.5 \| \| Several days \| 17 \| 34.0 \| 12 \| 24.5 \| 12 \| 30.0 \| 7 \| 24.1 \| \| More than half the days \| 8 \| 16.0 \| 5 \| 10.2 \| 1 \| 2.5 \| 1 \| 3.4 \| \| Nearly every day \| 11 \| 22.0 \| 4 \| 8.2 \| 3 \| 7.5 \| 2 \| 6.9 \| \| **Moving or speaking so slowly that other people could have noticed. Or the opposite—being so fidgety or restless that you have been moving around a lot more than usual** \| Not at all \| 22 \| 44.0 \| 34 \| 69.4 \| 29 \| 72.5 \| 25 \| 86.2 \| \| Several days \| 13 \| 26.0 \| 12 \| 24.5 \| 10 \| 25.0 \| 3 \| 10.3 \| \| More than half the days \| 8 \| 16.0 \| 2 \| 4.1 \| 1 \| 2.5 \| 0 \| 0.0 \| \| Nearly every day \| 7 \| 14.0 \| 1 \| 2.0 \| 0 \| 0.0 \| 1 \| 3.4 \| \| **Thoughts that you would be better off dead or of hurting yourself in some way** \| Not at all \| 43 \| 86.0 \| 48 \| 98.0 \| 38 \| 95.0 \| 29 \| 100 \| \| Several days \| 4 \| 8.0 \| 1 \| 2.0 \| 1 \| 2.5 \| 0 \| 0.0 \| \| More than half the days \| 2 \| 4.0 \| 0 \| 0.0 \| 1 \| 2.5 \| 0 \| 0.0 \| \| Nearly every day \| 1 \| 2.0 \| 0 \| 0.0 \| 0 \| 0.0 \| 0 \| 0.0 \| \| **If you checked off any problems, how difficult have these problems made it for you to do your work, take care of things at home, or get along with other people?** \| Not difficult at all \| 9 \| 18.0 \| 14 \| 28.6 \| 13 \| 32.5 \| 13 \| 44.8 \| \| Somewhat difficult \| 24 \| 48.0 \| 27 \| 55.1 \| 19 \| 47.5 \| 13 \| 44.8 \| \| Very difficult \| 14 \| 28.0 \| 6 \| 12.2 \| 1 \| 2.5 \| 0 \| 0.0 \| \| Extremely difficult \| 3 \| 6.0 \| 0 \| 0.0 \| 2 \| 5.0 \| 0 \| 0.0 \| \| Not applicable \| 0 \| 0.0 \| 2 \| 4.1 \| 5 \| 12.5 \| 3 \| 10.3 \| \| **PHQ Overall Scores** \|  \|  \|  \|  \|  \|  \|  \|  \|  \| \| **PHQ Severity** \| Minimal, 0–4 \| 6.0 \| 12.0 \| 20.0 \| 41 \| 20.0 \| 50.0 \| 17.0 \| 58.6 \| \| Mild, 5–9 \| 18.0 \| 36.0 \| 21.0 \| 43 \| 16.0 \| 40.0 \| 11.0 \| 37.9 \| \| Moderate, 10–14 \| 18.0 \| 36.0 \| 4.0 \| 8 \| 3.0 \| 7.5 \| 0.0 \| 0.0 \| \| Moderately severe, 15–19 \| 5.0 \| 10.0 \| 3.0 \| 6 \| 1.0 \| 2.5 \| 1.0 \| 3.4 \| \| Severe, 20–27 \| 3.0 \| 6.0 \| 1.0 \| 2 \| 0.0 \| 0.0 \| 0.0 \| 0.0 \| \| **PHQ Total Score** \| Mean, SD \| 10.6 \| 5.1 \| 5.8 \| 4.9 \| 4.7 \| 3.9 \| 4.2 \| 3.4 \| \| Median, IQR \| 10.0 \| 6.0 \| 5.0 \| 5.0 \| 4.5 \| 4.0 \| 4.0 \| 4.0 \| \| Minimum, maximum \| 2.0 \| 24.0 \| 0.0 \| 23.0 \| 0.0 \| 19.0 \| 0.0 \| 16.0 \| \| **PHQ Total Score 3 Month Change*** \| t-score (df), *p* value \| t = 4.9 (97) \| \| \| *p* < 0.0001 \| \|  \| \| \| \| **PHQ Total Score 6 Month Change*** \| t-score (df), *p* value \| t = 6.3 (87.9) \| \| \| *p* < 0.0001 \| \| \| **PHQ Total Score 12 Month Change*** \| t-score (df), *p* value \| t = 6.7 (75.4) \| \| \| *p* < 0.0001 \| \| \| **PHQ Total Score 3 to 6 Month Change** \| t-score (df), *p* value \| t = 1.3 (87) \| \| \| *p* = 0.215 \| \| \| **PHQ Total Score 6 to 12 Month Change** \| t-score (df), *p* value \| t = 0.5 (64.8) \| \| \| *p* = 0.645 \| \| |
| --- | --- | --- | --- | --- | --- | --- | --- | --- | --- | --- | --- | --- | --- | --- | --- | --- | --- | --- | --- | --- | --- | --- | --- | --- | --- | --- | --- | --- | --- | --- | --- | --- | --- | --- | --- | --- | --- | --- | --- | --- | --- | --- | --- | --- | --- | --- | --- | --- | --- | --- | --- | --- | --- | --- | --- | --- | --- | --- | --- | --- | --- | --- | --- | --- | --- | --- | --- | --- | --- | --- | --- | --- | --- | --- | --- | --- | --- | --- | --- | --- | --- | --- | --- | --- | --- | --- | --- | --- | --- | --- | --- | --- | --- | --- | --- | --- | --- | --- | --- | --- | --- | --- | --- | --- | --- | --- | --- | --- | --- | --- | --- | --- | --- | --- | --- | --- | --- | --- | --- | --- | --- | --- | --- | --- | --- | --- | --- | --- | --- | --- | --- | --- | --- | --- | --- | --- | --- | --- | --- | --- | --- | --- | --- | --- | --- | --- | --- | --- | --- | --- | --- | --- | --- | --- | --- | --- | --- | --- | --- | --- | --- | --- | --- | --- | --- | --- | --- | --- | --- | --- | --- | --- | --- | --- | --- | --- | --- | --- | --- | --- | --- | --- | --- | --- | --- | --- | --- | --- | --- | --- | --- | --- | --- | --- | --- | --- | --- | --- | --- | --- | --- | --- | --- | --- | --- | --- | --- | --- | --- | --- | --- | --- | --- | --- | --- | --- | --- | --- | --- | --- | --- | --- | --- | --- | --- | --- | --- | --- | --- | --- | --- | --- | --- | --- | --- | --- | --- | --- | --- | --- | --- | --- | --- | --- | --- | --- | --- | --- | --- | --- | --- | --- | --- | --- | --- | --- | --- | --- | --- | --- | --- | --- | --- | --- | --- | --- | --- | --- | --- | --- | --- | --- | --- | --- | --- | --- | --- | --- | --- | --- | --- | --- | --- | --- | --- | --- | --- | --- | --- | --- | --- | --- | --- | --- | --- | --- | --- | --- | --- | --- | --- | --- | --- | --- | --- | --- | --- | --- | --- | --- | --- | --- | --- | --- | --- | --- | --- | --- | --- | --- | --- | --- | --- | --- | --- | --- | --- | --- | --- | --- | --- | --- | --- | --- | --- | --- | --- | --- | --- | --- | --- | --- | --- | --- | --- | --- | --- | --- | --- | --- | --- | --- | --- | --- | --- | --- | --- | --- | --- | --- | --- | --- | --- | --- | --- | --- | --- | --- | --- | --- | --- | --- | --- | --- | --- | --- | --- | --- | --- | --- | --- | --- | --- | --- | --- | --- | --- | --- | --- | --- | --- | --- | --- | --- | --- | --- | --- | --- | --- | --- | --- | --- | --- | --- | --- | --- | --- | --- | --- | --- | --- | --- | --- | --- | --- | --- | --- | --- | --- | --- | --- | --- | --- | --- | --- | --- | --- | --- | --- | --- | --- | --- | --- | --- | --- | --- | --- | --- | --- | --- | --- | --- | --- | --- | --- | --- | --- | --- | --- | --- | --- | --- | --- | --- | --- | --- | --- | --- | --- | --- | --- | --- | --- | --- | --- | --- | --- | --- | --- | --- | --- | --- | --- | --- | --- | --- | --- | --- | --- | --- | --- | --- | --- | --- | --- | --- | --- | --- | --- | --- | --- | --- | --- | --- | --- | --- | --- | --- | --- | --- | --- | --- | --- | --- | --- | --- | --- | --- | --- | --- | --- | --- | --- | --- | --- | --- | --- | --- | --- |

*Change from baseline value.

*Note:* t-scores are calculated based on respondents with available data at all times points; the t-score is the ratio of differences between 2 timepoints.

df, degrees of freedom; IQR, interquartile range; PHQ 9, Patient Health Questionnaire 9; SD, standard deviation.

**Supplemental Table 2. WPAI:SHP Full Results**

| **Item Responses** |  | **Baseline (n = 50)** | | **Month 3 (n = 49)** | | **Month 6 (n = 40)** | | **Month 12 (n = 29)** | |
| --- | --- | --- | --- | --- | --- | --- | --- | --- | --- |
|  |  | **n** | **%** | **n** | **%** | **n** | **%** | **n** | **%** |
| **Are you currently employed (working for pay)?** | No | 22 | 44.0 | 21 | 42.9 | 18 | 45.0 | 17 | 58.6 |
|  | Yes | 28 | 56.0 | 28 | 57.1 | 22 | 55.0 | 12 | 41.4 |
| **During the past 7 days, how many hours did you miss from work because of problems associated with your HPP?** | Mean, SD | 1.8 | 3.8 | 0.3 | 1.5 | 0.0 | 0.0 | 2.9 | 9.8 |
|  | Median, IQR | 0.0 | 1.3 | 0.0 | 0.0 | 0.0 | 0.0 | 0.0 | 0.0 |
|  | Minimum, maximum | 0.0 | 15.0 | 0.0 | 8.0 | 0.0 | 0.0 | 0.0 | 40.0 |
| **3 Month Change*** | t-score (df), *p* value | t = 1.8 (35.3) | | *p* = 0.074 | |  | | | |
| **6 Month Change*** | t-score (df), *p* value | t = 2.4 (27) | | *p* = 0.022 | |  |  |  |  |
| **12 Month Change*** | t-score (df), *p* value | t = -0.46 (19) | | *p* = 0.652 | |  |  |  |  |
| **3 to 6 Month Change** | t-score (df), *p* value | t = 1.1 (27) | | *p* = 0.272 | |  |  |  |  |
| **6 to 12 Month Change** | t-score (df), *p* value | t = -1.22 (16) | | *p* = 0.241 | |  |  |  |  |
| **During the past 7 days, how many hours did you miss from work because of any other reason, such as vacation, holidays, time off to participate in this study?** | Mean, SD | 1.0 | 2.4 | 1.6 | 6.2 | 3.1 | 9.2 | 3.5 | 9.7 |
|  | Median, IQR | 0.0 | 0.0 | 0.0 | 0.0 | 0.0 | 0.8 | 0.0 | 1.0 |
|  | Minimum, maximum | 0.0 | 8.0 | 0.0 | 32.0 | 0.0 | 41.0 | 0.0 | 40.0 |
| **3 Month Change*** | t-score (df), *p* value | t = −0.5 (34.8) | | *p* = 0.649 | |  | | | |
| **6 Month Change*** | t-score (df), *p* value | t = −1 (23.2) | | *p* = 0.309 | |  |  |  |  |
| **12 Month Change*** | t-score (df), *p* value | t = −1 (17.2) | | *p* = 0.316 | |  |  |  |  |
| **3 to 6 Month Change** | t-score (df), *p* value | t = −0.7 (35) | | *p* = 0.509 | |  |  |  |  |
| **6 to 12 Month Change** | t-score (df), *p* value | t = −0.1 (33.6) | | *p* = 0.902 | |  |  |  |  |
| **During the past 7 days, how many hours did you actually work?** | Mean, SD | 33.9 | 10.2 | 30.2 | 14.3 | 34.7 | 7.7 | 29.2 | 14.3 |
|  | Median, IQR | 40.0 | 10.0 | 40.0 | 20.0 | 40.0 | 9.3 | 35.0 | 16.0 |
|  | Minimum, maximum | 0.0 | 41.0 | 0.0 | 41.0 | 15.0 | 41.0 | 0.0 | 41.0 |
| **3 Month Change*** | t-score (df), *p* value | t = 1.1 (48.8) | | *p* = 0.263 | |  | | | |
| **6 Month Change*** | t-score (df), *p* value | t = -0.3 (48) | | *p* = 0.753 | |  |  |  |  |
| **12 Month Change*** | t-score (df), *p* value | t = 1.2 (25.9) | | *p* = 0.247 | |  |  |  |  |
| **3 to 6 Month Change** | t-score (df), *p* value | t = -1.4 (43.1) | | *p* = 0.16 | |  |  |  |  |
| **6 to 12 Month Change** | t-score (df), *p* value | t = 1.4 (23.1) | | *p* = 0.166 | |  |  |  |  |
| **During the past 7 days, how much did your HP*P* affect your productivity while you were working?** | Mean, SD | 4.0 | 1.9 | 2.8 | 2.8 | 1.4 | 1.8 | 1.4 | 1.7 |
|  | Median, IQR | 4.0 | 2.0 | 3.0 | 5.5 | 0.0 | 3.0 | 1.0 | 2.5 |
|  | Minimum, maximum | 0.0 | 7.0 | 0.0 | 8.0 | 0.0 | 6.0 | 0.0 | 5.0 |
| **3 Month Change*** | t-score (df), *p* value | t = 1.8 (45.4) | | *p* = 0.086 | |  | | | |
| **6 Month Change*** | t-score (df), *p* value | t = 4.8 (45.5) | | *p* < 0.0001 | |  |  |  |  |
| **12 Month Change*** | t-score (df), *p* value | t = 4.5 (32.1) | | *p* < 0.0001 | |  |  |  |  |
| **3 to 6 Month Change** | t-score (df), *p* value | t = 2.1 (45) | | *p* = 0.042 | |  |  |  |  |
| **6 to 12 Month Change** | t-score (df), *p* value | t = 0.02 (32) | | *p* = 0.988 | |  |  |  |  |
| **During the past 7 days, not including today, how much did your HPP affect your ability to do your regular daily activities?** | Mean, SD | 6.4 | 2.1 | 4.2 | 2.4 | 2.9 | 2.4 | 3.0 | 2.7 |
|  | Median, IQR | 7.0 | 3.0 | 4.0 | 3.0 | 2.0 | 3.3 | 2.0 | 4.0 |
|  | Minimum, maximum | 0.0 | 10.0 | 0.0 | 8.0 | 0.0 | 8.0 | 0.0 | 10.0 |
| **3 Month Change*** | t-score (df), *p* value | t = 4.8 (93.5) | | *p* < 0.0001 | |  | | | |
| **6 Month Change*** | t-score (df), *p* value | t = 7.4 (76.3) | | *p* < 0.0001 | |  |  |  |  |
| **12 Month Change*** | t-score (df), *p* value | t = 5.8 (46.2) | | *p* < 0.0001 | |  |  |  |  |
| **3 to 6 Month Change** | t-score (df), *p* value | t = 2.7 (83.6) | | *p* = 0.01 | |  |  |  |  |
| **6 to 12 Month Change** | t-score (df), *p* value | t = −0.2 (55.8) | | *p* = 0.845 | |  |  |  |  |
| **Domains** |  |  |  |  |  |  |  |  |  |
| **Absenteeism (work time missed)†** | Mean , SD | 4.7 | 10.4 | 1.2 | 5.5 | 0.0 | 0.0 | 8.0 | 25.0 |
|  | Median, IQR | 0.0 | 3.8 | 0.0 | 0.0 | 0.0 | 0.0 | 0.0 | 0.0 |
|  | Minimum, maximum | 0.0 | 44.1 | 0.0 | 28.6 | 0.0 | 0.0 | 0.0 | 100 |
| **3 Month Change*** | t-score (df), *p* value | t = 1.6 (39.6) | | *p* = 0.121 | |  | | | |
| **6 Month Change*** | t-score (df), *p* value | t = 2.4 (26) | | *p* = 0.025 | |  |  |  |  |
| **12 Month Change*** | t-score (df), *p* value | t = −0.5 (18.1) | | *p* = 0.652 | |  |  |  |  |
| **3 to 6 Month Change** | t-score (df), *p* value | t = 1.1 (26) | | *p* = 0.288 | |  |  |  |  |
| **6 to 12 Month Change** | t-score (df), *p* value | t = −1.2 (15) | | *p* = 0.236 | |  |  |  |  |
| **Presenteeism (impairment at work/reduced on-the-job effectiveness)†** | Mean , SD | 39.6 | 18.9 | 28.2 | 28.3 | 14.1 | 18.4 | 14.0 | 17.0 |
|  | Median, IQR | 40.0 | 20.0 | 30.0 | 55.0 | 0.0 | 30.0 | 10.0 | 25.0 |
|  | Minimum, maximum | 0.0 | 70.0 | 0.0 | 80.0 | 0.0 | 60.0 | 0.0 | 50.0 |
| **3 Month Change*** | t-score (df), *p* value | t = 1.8 (45.4) | | *p* = 0.086 | |  | | | |
| **6 Month Change*** | t-score (df), *p* value | t = 4.8 (45.5) | | *p* < 0.0001 | |  |  |  |  |
| **12 Month Change*** | t-score (df), *p* value | t = 4.5 (32.1) | | *p* < 0.0001 | |  |  |  |  |
| **3 to 6 Month Change** | t-score (df), *p* value | t = 2.1 (45) | | *p* = 0.042 | |  |  |  |  |
| **6 to 12 Month Change** | t-score (df), *p* value | t = 0.02 (32) | | *p* = 0.988 | |  |  |  |  |
| **Activity impairment (activity impairment due to problems)†** | Mean , SD | 64.0 | 20.4 | 42.5 | 24.3 | 29.0 | 24.3 | 30.0 | 27.0 |
|  | Median, IQR | 70.0 | 30.0 | 40.0 | 30.0 | 20.0 | 33.0 | 20.0 | 4.0 |
|  | Minimum, maximum | 0 | 100 | 0 | 80.0 | 0 | 80.0 | 0 | 100 |
| **3 Month Change*** | t-score (df), *p* value | t = 4.8 (93.5) | | *p* < 0.0001 | |  | | | |
| **6 Month Change*** | t-score (df), *p* value | t = 7.5 (78.2) | | *p* < 0.0001 | |  |  |  |  |
| **12 Month Change*** | t-score (df), *p* value | t = 5.8 (46.2) | | *p* < 0.0001 | |  |  |  |  |
| **3 to 6 Month Change** | t-score (df), *p* value | t = 2.8 (85.2) | | *p* = 0.0063 | |  |  |  |  |
| **6 to 12 Month Change** | t-score (df), *p* value | t = -0.3 (55.8) | | *p* = 0.760 | |  |  |  |  |
| **Work productivity loss (overall work impairment/absenteeism plus presenteeism)†** | Mean , SD | 41.9 | 20.8 | 28.6 | 28.9 | 14.1 | 18.4 | 20.0 | 28.0 |
|  | Median, IQR | 40.0 | 29.2 | 30.0 | 55.0 | 0.0 | 30.0 | 10.0 | 30.0 |
|  | Minimum, maximum | 0.0 | 77.7 | 0.0 | 80.0 | 0.0 | 60.0 | 0.0 | 100 |
| **3 Month Change*** | t-score (df), *p* value | t = 1.9 (47.2) | | *p* = 0.059 | |  | | | |
| **6 Month Change*** | t-score (df), *p* value | t = 5.0 (46.6) | | *p* < 0.0001 | |  |  |  |  |
| **12 Month Change*** | t-score (df), *p* value | t = 2.7 (25.1) | | *p* = 0.012 | |  |  |  |  |
| **3 to 6 Month Change** | t-score (df), *p* value | t = 2.1 (44.7) | | *p* = 0.039 | |  |  |  |  |
| **6 to 12 Month Change** | t-score (df), *p* value | t = -0.78 (24.4) | | *p* = 0.446 | |  |  |  |  |

*Change from baseline value.

†Outcomes are expressed as impairment percentages, with higher numbers indicating greater impairment and less productivity (i.e., worse outcomes).

*Note*: t-scores are calculated based on respondents with available data at all times points; the t-score is the ratio of differences between 2 timepoints. Negative t-scores indicate that the average score is larger for the more recent timepoint.

df, degrees of freedom; IQR, interquartile range; SD, standard deviation; WPAI:SHP, Work Productivity and Activity Impairment–Specific Health Problem.

**Supplemental Table 3. PROMIS-29 Full Results**

|  | | **Baseline (n = 50)** | | **Month 3 (n = 49)** | | **Month 6 (n = 40)** | | **Month 12 (n = 29)** | |
| --- | --- | --- | --- | --- | --- | --- | --- | --- | --- |
|  |  | **n** | **%** | **n** | **%** | **n** | **%** | **n** | **%** |
| **PROMIS administered** |  | 50 | 100 | 49 | 98.0 | 40 | 90.9 | 29 | 100 |
| **Physical Function** |  |  |  |  |  |  |  |  |  |
| **Able to do chores such as vacuuming or housework** | Without any difficulty | 7 | 14.0 | 15 | 30.6 | 15 | 37.5 | 15 | 51.7 |
|  | With a little difficulty | 7 | 14.0 | 10 | 20.4 | 11 | 27.5 | 6 | 20.7 |
|  | With some difficulty | 23 | 46.0 | 18 | 36.7 | 11 | 27.5 | 5 | 17.2 |
|  | With much difficulty | 11 | 22.0 | 5 | 10.2 | 2 | 5.0 | 3 | 10.3 |
|  | Unable to do | 2 | 4.0 | 1 | 2.0 | 1 | 2.5 | 0 | 0.0 |
| **Able to go up and downs stairs at a normal pace** | Without any difficulty | 6 | 12.0 | 19 | 38.8 | 15 | 37.5 | 12 | 41.4 |
|  | With a little difficulty | 6 | 12.0 | 7 | 14.3 | 9 | 22.5 | 9 | 31.0 |
|  | With some difficulty | 22 | 44.0 | 15 | 30.6 | 13 | 32.5 | 6 | 20.7 |
|  | With much difficulty | 15 | 30.0 | 7 | 14.3 | 3 | 7.5 | 1 | 3.4 |
|  | Unable to do | 1 | 2.0 | 1 | 2.0 | 0 | 0.0 | 1 | 3.4 |
| **Able to go for a walk of at least 15 minutes** | Without any difficulty | 15 | 30.0 | 22 | 44.9 | 20 | 50.0 | 18 | 62.1 |
|  | With a little difficulty | 9 | 18.0 | 4 | 8.2 | 9 | 22.5 | 5 | 17.2 |
|  | With some difficulty | 11 | 22.0 | 16 | 32.7 | 9 | 22.5 | 3 | 10.3 |
|  | With much difficulty | 9 | 18.0 | 5 | 10.2 | 0 | 0.0 | 1 | 3.4 |
|  | Unable to do | 6 | 12.0 | 2 | 4.1 | 2 | 5.0 | 2 | 6.9 |
| **Able to run errands and shop** | Without any difficulty | 7 | 14.0 | 20 | 40.8 | 18 | 45.0 | 16 | 55.2 |
|  | With a little difficulty | 14 | 28.0 | 9 | 18.4 | 11 | 27.5 | 8 | 27.6 |
|  | With some difficulty | 18 | 36.0 | 17 | 34.7 | 9 | 22.5 | 3 | 10.3 |
|  | With much difficulty | 9 | 18.0 | 2 | 4.1 | 2 | 5.0 | 2 | 6.9 |
|  | Unable to do | 2 | 4.0 | 1 | 2.0 | 0 | 0.0 | 0 | 0.0 |
| **Physical Function Overall Score**^a^ | Mean, SD | 38.0 | 5.8 | 43.0 | 8.4 | 44.6 | 8.3 | 46.5 | 8.7 |
|  | Median, IQR | 37.2 | 6.1 | 40.2 | 11.5 | 42.0 | 12.3 | 45.0 | 16.8 |
|  | Minimum, maximum | 22.6 | 57.0 | 26.9 | 57.0 | 28.9 | 57.0 | 31.0 | 56.9 |
| **Physical Functional 3 Month Change*** | t-score (df), *p* value | t = -3.5 (85.2) | | *p* = 0.001 | |  | | | |
| **Physical Functional 6 Month Change*** | t-score (df), *p* value | t = -4.3 (67.6) | | *p* < 0.0001 | |  |  |  |  |
| **Physical Functional 12 Month Change*** | t-score (df), *p* value | t = -4.7 (42.9) | | *p* < 0.0001 | |  |  |  |  |
| **Physical Functional 3 to 6 Month Change** | t-score (df), *p* value | t = -0.9 (84.0) | | *p* = 0.3765 | |  |  |  |  |
| **Physical Functional 6 to 12 Month Change** | t-score (df), *p* value | t = -0.9 (58.8) | | *p* = 0.360 | |  |  |  |  |
| ^a^ Higher scores reflect greater physical functioning. Values are calculated based on standardized t-scores. | | | | | | | |  |  |
| **Anxiety** |  |  |  |  |  |  |  |  |  |
| **I felt fearful** | Never | 23 | 46.0 | 34 | 69.4 | 27 | 67.5 | 22 | 75.9 |
|  | Rarely | 6 | 12.0 | 8 | 16.3 | 8 | 20.0 | 5 | 17.2 |
|  | Sometimes | 12 | 24.0 | 4 | 8.2 | 3 | 7.5 | 2 | 6.9 |
|  | Often | 9 | 18.0 | 3 | 6.1 | 2 | 5.0 | 0 | 0.0 |
|  | Always | 0 | 0.0 | 0 | 0.0 | 0 | 0.0 | 0 | 0.0 |
| **I found it hard to focus on anything other than my anxiety** | Never | 17 | 34.0 | 26 | 53.1 | 25 | 62.5 | 24 | 82.8 |
|  | Rarely | 11 | 22.0 | 13 | 26.5 | 10 | 25.0 | 4 | 13.8 |
|  | Sometimes | 17 | 34.0 | 6 | 12.2 | 4 | 10.0 | 1 | 3.4 |
|  | Often | 4 | 8.0 | 4 | 8.2 | 0 | 0.0 | 0 | 0.0 |
|  | Always | 1 | 2.0 | 0 | 0.0 | 1 | 2.5 | 0 | 0.0 |
| **My worries overwhelmed me** | Never | 12 | 24.0 | 28 | 57.1 | 25 | 62.5 | 17 | 58.6 |
|  | Rarely | 15 | 30.0 | 11 | 22.4 | 10 | 25.0 | 7 | 24.1 |
|  | Sometimes | 16 | 32.0 | 7 | 14.3 | 4 | 10.0 | 5 | 17.2 |
|  | Often | 6 | 12.0 | 3 | 6.1 | 1 | 2.5 | 0 | 0.0 |
|  | Always | 1 | 2.0 | 0 | 0.0 | 0 | 0.0 | 0 | 0.0 |
| **I felt uneasy** | Never | 9 | 18.0 | 16 | 32.7 | 20 | 50.0 | 14 | 48.3 |
|  | Rarely | 14 | 28.0 | 23 | 46.9 | 12 | 30.0 | 11 | 37.9 |
|  | Sometimes | 17 | 34.0 | 7 | 14.3 | 7 | 17.5 | 3 | 10.3 |
|  | Often | 7 | 14.0 | 2 | 4.1 | 1 | 2.5 | 1 | 3.4 |
|  | Always | 3 | 6.0 | 1 | 2.0 | 0 | 0.0 | 0 | 0.0 |
| **Anxiety Overall Score**^b^ | Mean, SD | 57.5 | 8.9 | 51.5 | 8.7 | 49.4 | 8.0 | 47.9 | 7.3 |
|  | Median, IQR | 55.9 | 11.6 | 51.4 | 6.2 | 48.4 | 14.0 | 48.2 | 11.5 |
|  | Minimum, maximum | 40.3 | 73.3 | 40.3 | 73.4 | 40.3 | 70.7 | 40.3 | 63.5 |
| **Anxiety 3 Month Change*** | t-score (df), *p* value | t = 3.4 (97.0) | | *p* = 0.0011 | |  | | | |
| **Anxiety 6 Month Change*** | t-score (df), *p* value | t = 4.5 (86.8) | | *p* < 0.0001 | |  |  |  |  |
| **Anxiety 12 Month Change*** | t-score (df), *p* value | t = 5.1 (67.8) | | *p* < 0.0001 | |  |  |  |  |
| **Anxiety 3 to 6 Month Change** | t-score (df), *p* value | t = 1.2 (85.8) | | *p* = 0.2381 | |  |  |  |  |
| **Anxiety 6 to 12 Month Change** | t-score (df), *p* value | t = 0.8 (63.2) | | *p* = 0.435 | |  |  |  |  |
| ^b^ Higher scores reflect more severe levels of anxiety symptoms. Values are calculated based on standardized t-scores. | | | | | | | |  |  |
| **Depression** |  |  |  |  |  |  |  |  |  |
| **I felt worthless** | Never | 29 | 58.0 | 34 | 69.4 | 34 | 85.0 | 23 | 79.3 |
|  | Rarely | 10 | 20.0 | 10 | 20.4 | 1 | 2.5 | 5 | 17.2 |
|  | Sometimes | 10 | 20.0 | 2 | 4.1 | 5 | 12.5 | 1 | 3.4 |
|  | Often | 1 | 2.0 | 3 | 6.1 | 0 | 0.0 | 0 | 0.0 |
|  | Always | 0 | 0.0 | 0 | 0.0 | 0 | 0.0 | 0 | 0.0 |
| **I felt helpless** | Never | 24 | 48.0 | 29 | 59.2 | 28 | 70.0 | 20 | 69.0 |
|  | Rarely | 9 | 18.0 | 9 | 18.4 | 5 | 12.5 | 5 | 17.2 |
|  | Sometimes | 11 | 22.0 | 7 | 14.3 | 6 | 15.0 | 4 | 13.8 |
|  | Often | 6 | 12.0 | 4 | 8.2 | 1 | 2.5 | 0 | 0.0 |
|  | Always | 0 | 0.0 | 0 | 0.0 | 0 | 0.0 | 0 | 0.0 |
| **I felt depressed** | Never | 18 | 36.0 | 25 | 51.0 | 27 | 67.5 | 17 | 58.6 |
|  | Rarely | 11 | 22.0 | 12 | 24.5 | 8 | 20.0 | 8 | 27.6 |
|  | Sometimes | 14 | 28.0 | 8 | 16.3 | 4 | 10.0 | 4 | 13.8 |
|  | Often | 7 | 14.0 | 4 | 8.2 | 1 | 2.5 | 0 | 0.0 |
|  | Always | 0 | 0.0 | 0 | 0.0 | 0 | 0.0 | 0 | 0.0 |
| **I felt hopeless** | Never | 29 | 58.0 | 34 | 69.4 | 34 | 85.0 | 22 | 75.9 |
|  | Rarely | 11 | 22.0 | 10 | 20.4 | 3 | 7.5 | 6 | 20.7 |
|  | Sometimes | 5 | 10.0 | 2 | 4.1 | 2 | 5.0 | 1 | 3.4 |
|  | Often | 5 | 10.0 | 3 | 6.1 | 1 | 2.5 | 0 | 0.0 |
|  | Always | 0 | 0.0 | 0 | 0.0 | 0 | 0.0 | 0 | 0.0 |
| **Depression Overall Score**^c^ | Mean, SD | 52.6 | 8.4 | 49.8 | 8.4 | 46.6 | 7.4 | 47.4 | 6.7 |
|  | Median, IQR | 53.2 | 9.7 | 51.2 | 14.4 | 41.0 | 10.4 | 48.9 | 13.1 |
|  | Minimum, maximum | 41.0 | 69.5 | 41.0 | 69.5 | 41.0 | 65.8 | 41.0 | 58.9 |
| **Depression 3 Month Change*** | t-score (df), *p* value | t = 1.7 (97.0) | | *p* = 0.0897 | |  | | | |
| **Depression 6 Month Change*** | t-score (df), *p* value | t = 3.7 (87.0) | | *p* = 0.0005 | |  |  |  |  |
| **Depression 12 Month Change*** | t-score (df), *p* value | t = 3.1 (69.1) | | *p* = 0.003 | |  |  |  |  |
| **Depression 3 to 6 Month Change** | t-score (df), *p* value | t = 1.9 (86.3) | | *p* = 0.0593 | |  |  |  |  |
| **Depression 6 to 12 Month Change** | t-score (df), *p* value | t = -0.5 (63.7) | | *p* = 0.630 | |  |  |  |  |
| ^c^ Higher scores reflect more severe levels of depressive symptoms. Values are calculated based on standardized t-scores. | | | | | | | |  |  |
| **Fatigue** |  |  |  |  |  |  |  |  |  |
| **I feel fatigued** | Not at all | 0 | 0.0 | 3 | 6.1 | 3 | 7.5 | 4 | 13.8 |
|  | A little bit | 5 | 10.0 | 14 | 28.6 | 18 | 45.0 | 11 | 37.9 |
|  | Somewhat | 11 | 22.0 | 13 | 26.5 | 11 | 27.5 | 5 | 17.2 |
|  | Quite a bit | 14 | 28.0 | 10 | 20.4 | 5 | 12.5 | 7 | 24.1 |
|  | Very much | 20 | 40.0 | 9 | 18.4 | 3 | 7.5 | 2 | 6.9 |
| **I have trouble starting things because I am tired** | Not at all | 1 | 2.0 | 9 | 18.4 | 11 | 27.5 | 8 | 27.6 |
|  | A little bit | 7 | 14.0 | 15 | 30.6 | 15 | 37.5 | 12 | 41.4 |
|  | Somewhat | 14 | 28.0 | 13 | 26.5 | 10 | 25.0 | 6 | 20.7 |
|  | Quite a bit | 17 | 34.0 | 5 | 10.2 | 2 | 5.0 | 2 | 6.9 |
|  | Very much | 11 | 22.0 | 7 | 14.3 | 2 | 5.0 | 1 | 3.4 |
| **How run-down did you feel on average?** | Not at all | 1 | 2.0 | 7 | 14.3 | 7 | 17.5 | 4 | 13.8 |
|  | A little bit | 6 | 12.0 | 14 | 28.6 | 19 | 47.5 | 16 | 55.2 |
|  | Somewhat | 11 | 22.0 | 17 | 34.7 | 8 | 20.0 | 6 | 20.7 |
|  | Quite a bit | 21 | 42.0 | 8 | 16.3 | 5 | 12.5 | 2 | 6.9 |
|  | Very much | 11 | 22.0 | 3 | 6.1 | 1 | 2.5 | 1 | 3.4 |
| **How fatigued were you on average?** | Not at all | 0 | 0.0 | 4 | 8.2 | 3 | 7.5 | 5 | 17.2 |
|  | A little bit | 8 | 16.0 | 16 | 32.7 | 20 | 50.0 | 11 | 37.9 |
|  | Somewhat | 9 | 18.0 | 13 | 26.5 | 10 | 25.0 | 9 | 31.0 |
|  | Quite a bit | 21 | 42.0 | 15 | 30.6 | 5 | 12.5 | 3 | 10.3 |
|  | Very much | 12 | 24.0 | 1 | 2.0 | 2 | 5.0 | 1 | 3.4 |
| **Fatigue Overall Score**^d^ | Mean, SD | 63.3 | 8.0 | 55.3 | 9.9 | 51.9 | 8.8 | 51.3 | 8.5 |
|  | Median, IQR | 64.7 | 11.3 | 55.2 | 14.1 | 51.0 | 10.8 | 48.6 | 11.2 |
|  | Minimum, maximum | 46.0 | 75.8 | 33.7 | 75.8 | 33.7 | 75.8 | 33.7 | 66.6 |
| **Fatigue 3 Month Change*** | t-score (df), *p* value | t = 4.5 (92.2) | | *p* < 0.0001 | |  | | | |
| **Fatigue 6 Month Change*** | t-score (df), *p* value | t = 6.3 (79.6) | | *p* < 0.0001 | |  |  |  |  |
| **Fatigue 12 Month Change*** | t-score (df), *p* value | t = 6.1 (55.5) | | *p* < 0.0001 | |  |  |  |  |
| **Fatigue 3 to 6 Month Change** | t-score (df), *p* value | t = 1.7 (86.2) | | *p* = 0.0984 | |  |  |  |  |
| **Fatigue 6 to 12 Month Change** | t-score (df), *p* value | t = 0.3 (61.7) | | *p* = 0.7804 | |  |  |  |  |
| ^d^ Higher scores reflect greater levels of fatigue. Values are calculated based on standardized t-scores. | | | | | | | |  |  |
| **Sleep Disturbance** |  |  |  |  |  |  |  |  |  |
| **My sleep quality was** | Very poor | 10 | 20.0 | 4 | 8.2 | 2 | 5.0 | 1 | 3.4 |
|  | Poor | 19 | 38.0 | 9 | 18.4 | 8 | 20.0 | 5 | 17.2 |
|  | Fair | 14 | 28.0 | 24 | 49.0 | 17 | 42.5 | 10 | 34.5 |
|  | Good | 6 | 12.0 | 9 | 18.4 | 11 | 27.5 | 11 | 37.9 |
|  | Very good | 1 | 2.0 | 3 | 6.1 | 2 | 5.0 | 2 | 6.9 |
| **My sleep was refreshing** | Not at all | 14 | 28.0 | 14 | 28.6 | 7 | 17.5 | 3 | 10.3 |
|  | A little bit | 25 | 50.0 | 12 | 24.5 | 8 | 20.0 | 11 | 37.9 |
|  | Somewhat | 8 | 16.0 | 15 | 30.6 | 17 | 42.5 | 9 | 31.0 |
|  | Quite a bit | 3 | 6.0 | 6 | 12.2 | 8 | 20.0 | 5 | 17.2 |
|  | Very much | 0 | 0.0 | 2 | 4.1 | 0 | 0.0 | 1 | 3.4 |
| **I had a problem with my sleep** | Not at all | 3 | 6.0 | 11 | 22.4 | 10 | 25.0 | 6 | 20.7 |
|  | A little bit | 11 | 22.0 | 9 | 18.4 | 8 | 20.0 | 8 | 27.6 |
|  | Somewhat | 4 | 8.0 | 10 | 20.4 | 10 | 25.0 | 8 | 27.6 |
|  | Quite a bit | 17 | 34.0 | 12 | 24.5 | 9 | 22.5 | 5 | 17.2 |
|  | Very much | 15 | 30.0 | 7 | 14.3 | 3 | 7.5 | 2 | 6.9 |
| **I had difficulty falling asleep** | Not at all | 13 | 26.0 | 15 | 30.6 | 17 | 42.5 | 16 | 55.2 |
|  | A little bit | 11 | 22.0 | 13 | 26.5 | 11 | 27.5 | 3 | 10.3 |
|  | Somewhat | 9 | 18.0 | 6 | 12.2 | 5 | 12.5 | 6 | 20.7 |
|  | Quite a bit | 8 | 16.0 | 8 | 16.3 | 5 | 12.5 | 2 | 6.9 |
|  | Very much | 9 | 18.0 | 7 | 14.3 | 2 | 5.0 | 2 | 6.9 |
| **Sleep Disturbance Overall Score**^e^ | Mean, SD | 58.8 | 8.1 | 54.3 | 8.9 | 52.3 | 7.5 | 51.6 | 8.9 |
|  | Median, IQR | 59.0 | 9.1 | 53.0 | 11.2 | 52.8 | 9.0 | 51.7 | 9.9 |
|  | Minimum, maximum | 40.7 | 73.3 | 32.0 | 73.3 | 36.9 | 66.4 | 32.0 | 73.3 |
| **Sleep Disturbance 3 Month Change*** | t-score (df), *p* value | t = 2.6 (95.8) | | *p* = 0.0099 | |  | | | |
| **Sleep Disturbance 6 Month Change*** | t-score (df), *p* value | t = 3.9 (85.9) | | *p* = 0.0002 | |  |  |  |  |
| **Sleep Disturbance 12 Month Change*** | t-score (df), *p* value | t = 3.6 (54.0) | | *p* = 0.0008 | |  |  |  |  |
| **Sleep Disturbance 3 to 6 Month Change** | t-score (df), *p* value | t = 1.1 (86.8) | | *p* = 0.2632 | |  |  |  |  |
| **Sleep Disturbance 6 to 12 Month Change** | t-score (df), *p* value | t = 0.4 (54.1) | | *p* = 0.7236 | |  |  |  |  |
| ^e^ Higher scores reflect greater sleep disturbances. Values are calculated based on standardized t-scores. | | | | | |  |  |  |  |
| **Ability to Participate in Social Roles and Activities** | |  |  |  |  |  |  |  |  |
| **I have trouble doing all of my regular leisure activities with others** | Never | 5 | 10.0 | 11 | 22.4 | 13 | 32.5 | 10 | 34.5 |
|  | Rarely | 5 | 10.0 | 14 | 28.6 | 13 | 32.5 | 8 | 27.6 |
|  | Sometimes | 22 | 44.0 | 14 | 28.6 | 13 | 32.5 | 7 | 24.1 |
|  | Usually | 13 | 26.0 | 9 | 18.4 | 0 | 0.0 | 1 | 3.4 |
|  | Always | 5 | 10.0 | 1 | 2.0 | 1 | 2.5 | 3 | 10.3 |
| **I have trouble doing all of the family activities that I want to do** | Never | 3 | 6.0 | 8 | 16.3 | 12 | 30.0 | 9 | 31.0 |
|  | Rarely | 4 | 8.0 | 11 | 22.4 | 11 | 27.5 | 9 | 31.0 |
|  | Sometimes | 25 | 50.0 | 22 | 44.9 | 12 | 30.0 | 7 | 24.1 |
|  | Usually | 14 | 28.0 | 6 | 12.2 | 4 | 10.0 | 2 | 6.9 |
|  | Always | 4 | 8.0 | 2 | 4.1 | 1 | 2.5 | 2 | 6.9 |
| **I have trouble doing all of my usual work (include work at home)** | Never | 1 | 2.0 | 7 | 14.3 | 10 | 25.0 | 7 | 24.1 |
|  | Rarely | 8 | 16.0 | 15 | 30.6 | 13 | 32.5 | 9 | 31.0 |
|  | Sometimes | 27 | 54.0 | 18 | 36.7 | 12 | 30.0 | 11 | 37.9 |
|  | Usually | 9 | 18.0 | 6 | 12.2 | 3 | 7.5 | 2 | 6.9 |
|  | Always | 5 | 10.0 | 3 | 6.1 | 2 | 5.0 | 0 | 0.0 |
| **I have trouble doing all of the activities with friends that I want to do** | Never | 2 | 4.0 | 8 | 16.3 | 10 | 25.0 | 7 | 24.1 |
|  | Rarely | 6 | 12.0 | 13 | 26.5 | 11 | 27.5 | 9 | 31.0 |
|  | Sometimes | 25 | 50.0 | 15 | 30.6 | 14 | 35.0 | 11 | 37.9 |
|  | Usually | 9 | 18.0 | 12 | 24.5 | 2 | 5.0 | 1 | 3.4 |
|  | Always | 8 | 16.0 | 1 | 2.0 | 3 | 7.5 | 1 | 3.4 |
| **Social Roles and Activities Overall Score**^f^ | Mean, SD | 42.6 | 6.9 | 47.7 | 8.3 | 50.4 | 8.3 | 50.7 | 8.9 |
|  | Median, IQR | 43.3 | 5.8 | 46.3 | 9.5 | 50.2 | 7.7 | 50.2 | 9.6 |
|  | Minimum, maximum | 27.5 | 58.3 | 31.8 | 64.2 | 27.5 | 64.2 | 32.5 | 64.2 |
| **Social Roles 3 Month Change*** | t-score (df), *p* value | t = -3.3 (93.3) | | *p* = 0.0012 | |  | | | |
| **Social Roles 6 Month Change*** | t-score (df), *p* value | t = -4.8 (75.9) | | *p* < 0.0001 | |  |  |  |  |
| **Social Roles 12 Month Change*** | t-score (df), *p* value | t = -4.2 (47.9) | | *p* = 0.0001 | |  |  |  |  |
| **Social Roles 3 to 6 Month Change** | t-score (df), *p* value | t = -1.5 (83.6) | | *p* = 0.1345 | |  |  |  |  |
| **Social Roles 6 to 12 Month Change** | t-score (df), *p* value | t = -0.1 (58.0) | | *p* = 0.894 | |  |  |  |  |
| ^f^ Higher scores reflect greater functioning within social roles and activities. Values are calculated based on standardized t-scores. | | | | | | | |  |  |
| **Pain Interference** |  |  |  |  |  |  |  |  |  |
| **How much did pain interfere with your day to day activities?** | Not at all | 2 | 4.0 | 8 | 16.3 | 5 | 12.5 | 8 | 27.6 |
|  | A little bit | 4 | 8.0 | 8 | 16.3 | 14 | 35.0 | 7 | 24.1 |
|  | Somewhat | 12 | 24.0 | 14 | 28.6 | 13 | 32.5 | 10 | 34.5 |
|  | Quite a bit | 19 | 38.0 | 16 | 32.7 | 8 | 20.0 | 3 | 10.3 |
|  | Very much | 13 | 26.0 | 3 | 6.1 | 0 | 0.0 | 1 | 3.4 |
| **How did pain interfere with work around the home?** | Not at all | 4 | 8.0 | 12 | 24.5 | 7 | 17.5 | 9 | 31.0 |
|  | A little bit | 4 | 8.0 | 6 | 12.2 | 12 | 30.0 | 7 | 24.1 |
|  | Somewhat | 13 | 26.0 | 14 | 28.6 | 15 | 37.5 | 8 | 27.6 |
|  | Quite a bit | 19 | 38.0 | 16 | 32.7 | 5 | 12.5 | 4 | 13.8 |
|  | Very much | 10 | 20.0 | 1 | 2.0 | 1 | 2.5 | 1 | 3.4 |
| **How did pain interfere with your ability to participate in social activities?** | Not at all | 7 | 14.0 | 14 | 28.6 | 15 | 37.5 | 15 | 51.7 |
|  | A little bit | 8 | 16.0 | 10 | 20.4 | 14 | 35.0 | 6 | 20.7 |
|  | Somewhat | 15 | 30.0 | 14 | 28.6 | 6 | 15.0 | 5 | 17.2 |
|  | Quite a bit | 13 | 26.0 | 9 | 18.4 | 4 | 10.0 | 2 | 6.9 |
|  | Very much | 7 | 14.0 | 2 | 4.1 | 1 | 2.5 | 1 | 3.4 |
| **How much did pain interfere with your household chores?** | Not at all | 4 | 8.0 | 13 | 26.5 | 10 | 25.0 | 11 | 37.9 |
|  | A little bit | 5 | 10.0 | 6 | 12.2 | 14 | 35.0 | 6 | 20.7 |
|  | Somewhat | 15 | 30.0 | 18 | 36.7 | 10 | 25.0 | 6 | 20.7 |
|  | Quite a bit | 19 | 38.0 | 9 | 18.4 | 4 | 10.0 | 5 | 17.2 |
|  | Very much | 7 | 14.0 | 3 | 6.1 | 2 | 5.0 | 1 | 3.4 |
| **Pain Interference Overall Score**^g^ | Mean, SD | 63.8 | 6.8 | 58.4 | 8.7 | 56.7 | 7.1 | 54.9 | 9.1 |
|  | Median, IQR | 64.2 | 6.4 | 61.3 | 13.5 | 57.3 | 6.2 | 55.8 | 11.1 |
|  | Minimum, maximum | 41.6 | 75.6 | 41.6 | 75.6 | 41.6 | 69.4 | 41.6 | 75.6 |
| **Pain Interference 3 Month Change*** | t-score (df), *p* value | t = 3.5 (90.6) | | *p* = 0.001 | |  | | | |
| **Pain Interference 6 Month Change*** | t-score (df), *p* value | t = 4.8 (81.9) | | *p* < 0.0001 | |  |  |  |  |
| **Pain Interference 12 Month Change*** | t-score (df), *p* value | t = 4.6 (46.4) | | *p* < 0.0001 | |  |  |  |  |
| **Pain Interference 3 to 6 Month Change** | t-score (df), *p* value | t = 1.0 (87.0) | | *p* = 0.3169 | |  |  |  |  |
| **Pain Interference 6 to 12 Month Change** | t-score (df), *p* value | t = 0.9 (51.3) | | *p* = 0.3838 | |  |  |  |  |

*Change from baseline value.

df, degrees of freedom; IQR, interquartile range; PROMIS-29, Patient-Reported Outcomes Measurement Information System 29; SD, standard deviation.

**Supplemental Table 4. RAPID3 Full Results**

|  | | **Baseline (n = 50)** | | **Month 3 (n = 49)** | | **Month 6 (n = 40)** | | **Month 12 (n = 29)** | |
| --- | --- | --- | --- | --- | --- | --- | --- | --- | --- |
|  |  | **n** | **%** | **n** | **%** | **n** | **%** | **n** | **%** |
| **Over the last week were you able to…** |  |  |  |  |  |  |  |  |  |
| **Dress yourself, including tying shoelaces and doing buttons?** | Without any difficulty | 26 | 52.0 | 33 | 67.3 | 29 | 72.5 | 23 | 79.3 |
|  | With some difficulty | 21 | 42.0 | 14 | 28.6 | 9 | 22.5 | 6 | 20.7 |
|  | With much difficulty | 2 | 4.0 | 2 | 4.1 | 1 | 2.5 | 0 | 0.0 |
|  | Unable to do | 1 | 2.0 | 0 | 0.0 | 1 | 2.5 | 0 | 0.0 |
| **Get in and out of bed?** | Without any difficulty | 19 | 38.0 | 25 | 51.0 | 29 | 72.5 | 26 | 89.7 |
|  | With some difficulty | 26 | 52.0 | 23 | 46.9 | 9 | 22.5 | 3 | 10.3 |
|  | With much difficulty | 4 | 8.0 | 1 | 2.0 | 2 | 5.0 | 0 | 0.0 |
|  | Unable to do | 1 | 2.0 | 0 | 0.0 | 0 | 0.0 | 0 | 0.0 |
| **Lift a full cup or glass to your mouth?** | Without any difficulty | 41 | 82.0 | 45 | 91.8 | 38 | 95.0 | 28 | 96.6 |
|  | With some difficulty | 9 | 18.0 | 4 | 8.2 | 2 | 5.0 | 1 | 3.4 |
|  | With much difficulty | 0 | 0.0 | 0 | 0.0 | 0 | 0.0 | 0 | 0.0 |
|  | Unable to do | 0 | 0.0 | 0 | 0.0 | 0 | 0.0 | 0 | 0.0 |
| **Walk outdoors on flat ground?** | Without any difficulty | 23 | 46.0 | 28 | 57.1 | 28 | 70.0 | 23 | 79.3 |
|  | With some difficulty | 23 | 46.0 | 19 | 38.8 | 9 | 22.5 | 5 | 17.2 |
|  | With much difficulty | 4 | 8.0 | 2 | 4.1 | 2 | 5.0 | 1 | 3.4 |
|  | Unable to do | 0 | 0.0 | 0 | 0.0 | 1 | 2.5 | 0 | 0.0 |
| **Wash and dry your entire body?** | Without any difficulty | 27 | 54.0 | 35 | 71.4 | 31 | 77.5 | 24 | 82.8 |
|  | With some difficulty | 22 | 44.0 | 13 | 26.5 | 8 | 20.0 | 5 | 17.2 |
|  | With much difficulty | 0 | 0.0 | 1 | 2.0 | 1 | 2.5 | 0 | 0.0 |
|  | Unable to do | 1 | 2.0 | 0 | 0.0 | 0 | 0.0 | 0 | 0.0 |

| **Bend down to pick up clothing from the floor?** | Without any difficulty | 17 | 34.0 | 19 | 38.8 | 21 | 52.5 | 15 | 51.7 |
| --- | --- | --- | --- | --- | --- | --- | --- | --- | --- |
|  | With some difficulty | 24 | 48.0 | 27 | 55.1 | 15 | 37.5 | 14 | 48.3 |
|  | With much difficulty | 6 | 12.0 | 1 | 2.0 | 3 | 7.5 | 0 | 0.0 |
|  | Unable to do | 3 | 6.0 | 2 | 4.1 | 1 | 2.5 | 0 | 0.0 |
| **Turn regular faucets on and off?** | Without any difficulty | 47 | 94.0 | 48 | 98.0 | 37 | 92.5 | 29 | 100 |
|  | With some difficulty | 1 | 2.0 | 1 | 2.0 | 3 | 7.5 | 0 | 0.0 |
|  | With much difficulty | 1 | 2.0 | 0 | 0.0 | 0 | 0.0 | 0 | 0.0 |
|  | Unable to do | 1 | 2.0 | 0 | 0.0 | 0 | 0.0 | 0 | 0.0 |
| **Get in and out of a car, bus, train, or airplane?** | Without any difficulty | 18 | 36.0 | 28 | 57.1 | 24 | 60.0 | 21 | 72.4 |
|  | With some difficulty | 28 | 56.0 | 20 | 40.8 | 16 | 40.0 | 8 | 27.6 |
|  | With much difficulty | 4 | 8.0 | 1 | 2.0 | 0 | 0.0 | 0 | 0.0 |
|  | Unable to do | 0 | 0.0 | 0 | 0.0 | 0 | 0.0 | 0 | 0.0 |
| **Walk 2 miles or 3 kilometers, if you wish?** | Without any difficulty | 7 | 14.0 | 20 | 40.8 | 17 | 42.5 | 14 | 48.3 |
|  | With some difficulty | 15 | 30.0 | 11 | 22.4 | 15 | 37.5 | 8 | 27.6 |
|  | With much difficulty | 10 | 20.0 | 9 | 18.4 | 1 | 2.5 | 4 | 13.8 |
|  | Unable to do | 18 | 36.0 | 9 | 18.4 | 7 | 17.5 | 3 | 10.3 |
| **Participate in recreational activities and sports as you would like, if you wish** | Without any difficulty | 2 | 4.0 | 10 | 20.4 | 7 | 17.5 | 11 | 37.9 |
|  | With some difficulty | 17 | 34.0 | 17 | 34.7 | 19 | 47.5 | 9 | 31.0 |
|  | With much difficulty | 12 | 24.0 | 11 | 22.4 | 6 | 15.0 | 6 | 20.7 |
|  | Unable to do | 19 | 38.0 | 11 | 22.4 | 8 | 20.0 | 3 | 10.3 |
| **Get a good night’s sleep?** | Without any difficulty | 7 | 14.0 | 8 | 16.3 | 9 | 22.5 | 10 | 34.5 |
|  | With some difficulty | 15 | 30.0 | 23 | 46.9 | 21 | 52.5 | 12 | 41.4 |
|  | With much difficulty | 19 | 38.0 | 12 | 24.5 | 6 | 15.0 | 6 | 20.7 |
|  | Unable to do | 9 | 18.0 | 6 | 12.2 | 4 | 10.0 | 1 | 3.4 |

| **Deal with feelings of anxiety or being nervous?** | Without any difficulty | 20 | 40.0 | 29 | 59.2 | 20 | 50.0 | 20 | 69.0 |
| --- | --- | --- | --- | --- | --- | --- | --- | --- | --- |
|  | With some difficulty | 22 | 44.0 | 14 | 28.6 | 18 | 45.0 | 9 | 31.0 |
|  | With much difficulty | 8 | 16.0 | 6 | 12.2 | 2 | 5.0 | 0 | 0.0 |
|  | Unable to do | 0 | 0.0 | 0 | 0.0 | 0 | 0.0 | 0 | 0.0 |
| **Deal with feelings of depression or feeling blue?** | Without any difficulty | 23 | 46.0 | 30 | 61.2 | 26 | 65.0 | 23 | 79.3 |
|  | With some difficulty | 24 | 48.0 | 14 | 28.6 | 13 | 32.5 | 6 | 20.7 |
|  | With much difficulty | 3 | 6.0 | 5 | 10.2 | 1 | 2.5 | 0 | 0.0 |
|  | Unable to do | 0 | 0.0 | 0 | 0.0 | 0 | 0.0 | 0 | 0.0 |
| **RAPID3 Scores and Subscores** |  |  |  |  |  |  |  |  |  |
| **Functional Status (0-10)** | Mean, SD | 2.7 | 1.6 | 1.8 | 1.5 | 1.6 | 1.4 | 1.1 | 1.2 |
|  | Median, IQR | 2.9 | 2.4 | 1.7 | 2.0 | 1.3 | 2.0 | 1.0 | 2.0 |
|  | Minimum, maximum | 0.0 | 8.0 | 0.0 | 6.3 | 0.0 | 6.3 | 0.0 | 4.3 |
| **Functional Status 3 Month Change*** | t-score (df), *p* value | t = 2.8 (96.7) | | *p* = 0.007 | |  | |  | |
| **Functional Status 6 Month Change*** | t-score (df), *p* value | t = 3.5 (87.3) | | *p* = 0.001 | |  |  |  |  |
| **Functional Status 12 Month Change*** | t-score (df), *p* value | t = 5.0 (72.8) | | *p* < 0.0001 | |  |  |  |  |
| **Functional Status 3 to 6 Month Change** | t-score (df), *p* value | t = 0.8 (85.3) | | *p* = 0.402 | |  |  |  |  |
| **Functional Status 6 to 12 Month Change** | t-score (df), *p* value | t = 1.5 (65.5) | | *p* = 0.138 | |  |  |  |  |
| **Pain Tolerance (0-10)** | Mean, SD | 6.0 | 1.8 | 4.5 | 2.5 | 3.6 | 1.9 | 3.2 | 2.2 |
|  | Median, IQR | 6.0 | 2.0 | 5.0 | 3.0 | 3.8 | 2.6 | 3.0 | 4.0 |
|  | Minimum, maximum | 1.5 | 10.0 | 0.0 | 9.0 | 1.0 | 7.5 | 0.0 | 7.0 |
| **Pain Tolerance 3 Month Change*** | t-score (df), *p* value | t = 3.2 (87.8) | | *p* = 0.002 | |  | |  | |
| **Pain Tolerance 6 Month Change*** | t-score (df), *p* value | t = 5.9 (83.2) | | *p* < 0.0001 | |  |  |  |  |
| **Pain Tolerance 12 Month Change*** | t-score (df), *p* value | t = 5.8 (51.8) | | *p* < 0.0001 | |  |  |  |  |
| **Pain Tolerance 3 to 6 Month Change** | t-score (df), *p* value | t = 2.0 (86.3) | | *p* = 0.053 | |  |  |  |  |
| **Pain Tolerance 6 to 12 Month Change** | t-score (df), *p* value | t = 0.87 (55.4) | | *p* = 0.387 | |  |  |  |  |

| **Global Health Estimate (0-10)** | Mean (SD) | 5.1 | 2.1 | 4.1 | 2.3 | 3.1 | 2.3 | 2.7 | 2.2 |
| --- | --- | --- | --- | --- | --- | --- | --- | --- | --- |
|  | Median (IQR) | 5.0 | 2.9 | 4.0 | 3.5 | 2.8 | 4.0 | 3.0 | 3.0 |
|  | Minimum, maximum | 1.0 | 10.0 | 0.0 | 8.5 | 0.0 | 8.0 | 0.0 | 8.0 |
| **Global Health 3 Month Change*** | t-score (df), *p* value | t = 2.3 (96.2) | | *p* = 0.022 | |  | |  | |
| **Global Health 6 Month Change*** | t-score (df), *p* value | t = 4.4 (79.8) | | *p* < 0.0001 | |  |  |  |  |
| **Global Health 12 Month Change*** | t-score (df), *p* value | t = 4.8 (56.6) | | *p* < 0.0001 | |  |  |  |  |
| **Global Health 3 to 6 Month Change** | t-score (df), *p* value | t = 2.2 (82.5) | | *p* = 0.035 | |  |  |  |  |
| **Global Health 6 to 12 Month Change** | t-score (df), *p* value | t = 0.66 (62.3) | | *p* = 0.513 | |  |  |  |  |
| **RAPID-3 Cumulative Score (0-30)** | Mean, SD | 13.8 | 4.6 | 10.5 | 5.3 | 8.3 | 4.8 | 7.0 | 4.8 |
|  | Median, IQR | 14.0 | 4.5 | 10.3 | 8.3 | 7.6 | 6.7 | 7.0 | 7.7 |
|  | Minimum, maximum | 3.0 | 27.5 | 0.0 | 20.3 | 1.0 | 19.8 | 0.0 | 15.3 |
| **RAPID-3 Total Score 3 Month Change*** | t-score (df), *p* value | t = 3.3 (95.1) | | *p* = 0.002 | |  | |  | |
| **RAPID-3 Total Score 6 Month Change*** | t-score (df), *p* value | t = 5.4 (82.1) | | *p* < 0.0001 | |  |  |  |  |
| **RAPID-3 Total Score 12 Month Change*** | t-score (df), *p* value | t = 6.2 (56.7) | | *p* < 0.0001 | |  |  |  |  |
| **RAPID-3 Total Score 3 to 6 Month Change** | t-score (df), *p* value | t = 2.1 (85.6) | | *p* = 0.041 | |  |  |  |  |
| **RAPID-3 Total Score 6 to 12 Month Change** | t-score (df), *p* value | t = 1.1 (60.7) | | *p* = 0.288 | |  |  |  |  |
| **RAPID-3 Weighted Score (0.3-10.0)** | Mean, SD | 4.6 | 1.6 | 3.5 | 1.8 | 2.8 | 1.6 | 2.4 | 1.6 |
|  | Median, IQR | 4.7 | 1.5 | 3.3 | 2.7 | 2.7 | 2.4 | 2.3 | 2.7 |
|  | Minimum, maximum | 1.0 | 9.3 | 0.0 | 6.7 | 0.3 | 6.7 | 0.0 | 5.0 |
| **RAPID-3 Weighted Score 3 Month Change*** | t-score (df), *p* value | t = 3.3 (95.1) | | *p* = 0.0014 | |  | |  | |
| **RAPID-3 Weighted Score 6 Month Change*** | t-score (df), *p* value | t = 5.5 (82) | | *p* < 0.0001 | |  |  |  |  |
| **RAPID-3 Weighted Score 12 Month Change*** | t-score (df), *p* value | t = 6.1 (56.9) | | *p* < 0.0001 | |  |  |  |  |
| **RAPID-3 Weighted Score 3 to 6 Month Change** | t-score (df), *p* value | t = 2.1 (85.5) | | *p* = 0.041 | |  |  |  |  |
| **RAPID-3 Weighted Score 6 to 12 Month Change** | t-score (df), *p* value | t = 1.1 (60.8) | | *p* = 0.295 | |  |  |  |  |

| **RAPID Qualitative Description of Total Score** | Near Remission (0-1.0) | 2 | 4.0 | 6 | 12.2 | 7 | 17.5 | 8 | 27.6 |
| --- | --- | --- | --- | --- | --- | --- | --- | --- | --- |
|  | Low Severity (1.3-2.0) | 1 | 2.0 | 6 | 12.2 | 10 | 25.0 | 5 | 17.2 |
|  | Moderate Severity (2.3-4.0) | 15 | 30.0 | 21 | 42.9 | 16 | 40.0 | 11 | 37.9 |
|  | High Severity (4.3-10.0) | 32 | 64.0 | 16 | 32.7 | 7 | 17.5 | 5 | 17.2 |

*Change from baseline value.

df, degrees of freedom; IQR, interquartile range; RAPID3, Routine Assessment of Patient Index Data 3; standard deviation.

**Supplemental Table 5. TSQM Full Results**

|  | | **Month 12 (n = 29)** | |
| --- | --- | --- | --- |
|  |  | **n** | **%** |
| **Over the last 2 to 3 weeks, or since you last used your medication…** | |  |  |
| **How satisfied or dissatisfied are you with the ability of the medication to prevent or treat your condition?** | Extremely Dissatisfied | 0 | 0.0 |
|  | Very Dissatisfied | 1 | 3.4 |
|  | Dissatisfied | 1 | 3.4 |
|  | Somewhat Satisfied | 6 | 20.7 |
|  | Satisfied | 5 | 17.2 |
|  | Very Satisfied | 8 | 27.6 |
|  | Extremely Satisfied | 8 | 27.6 |
| **How satisfied or dissatisfied are you with the way the medication relieves your symptoms?** | Extremely Dissatisfied | 0 | 0.0 |
|  | Very Dissatisfied | 1 | 3.4 |
|  | Dissatisfied | 2 | 6.9 |
|  | Somewhat Satisfied | 7 | 24.1 |
|  | Satisfied | 5 | 17.2 |
|  | Very Satisfied | 8 | 27.6 |
|  | Extremely Satisfied | 6 | 20.7 |
| **How satisfied or dissatisfied are you with the amount of time it takes the medication to start working?** | Extremely Dissatisfied | 0 | 0.0 |
|  | Very Dissatisfied | 2 | 6.9 |
|  | Dissatisfied | 1 | 3.4 |
|  | Somewhat Satisfied | 6 | 20.7 |
|  | Satisfied | 8 | 27.6 |
|  | Very Satisfied | 9 | 31.0 |
|  | Extremely Satisfied | 3 | 10.3 |

| **As a result of taking this medication, do you experience any side effects at all?** | Yes | 13 | 44.8 |  |
| --- | --- | --- | --- | --- |
|  | No | 16 | 55.2 |  |
|  |  |  |  |  |
| **How bothersome are the side effects of the medication you take to treat your condition?*** | Extremely Bothersome | 2 | 15.4 |  |
|  | Very Bothersome | 0 | 0.0 |  |
|  | Somewhat Bothersome | 2 | 15.4 |  |
|  | A Little Bothersome | 8 | 61.5 |  |
|  | Not at All Bothersome | 1 | 7.7 |  |
| **To what extent do the side effects interfere with your physical health and ability to function (i.e., strength, energy levels, etc.)?*** | A Great Deal | 0 | 0.0 |  |
|  | Quite a Bit | 1 | 7.7 |  |
|  | Somewhat | 1 | 7.7 |  |
|  | Minimally | 1 | 7.7 |  |
|  | Not at All | 10 | 76.9 |  |
| **To what extent do the side effects interfere with your mental function (i.e., ability to think clearly, stay awake, etc.)?*** | A Great Deal | 1 | 7.7 |  |
|  | Quite a Bit | 1 | 7.7 |  |
|  | Somewhat | 1 | 7.7 |  |
|  | Minimally | 2 | 15.4 |  |
|  | Not at All | 8 | 61.5 |  |
| **To what degree have medication side effects affected your overall satisfaction with the medication?*** | A Great Deal | 0 | 0.0 |  |
|  | Quite a Bit | 1 | 7.7 |  |
|  | Somewhat | 3 | 23.1 |  |
|  | Minimally | 5 | 38.5 |  |
|  | Not at All | 4 | 30.8 |  |

| **How easy or difficult is it to use the medication in its current form?** | Extremely Difficult | 0 | 0.0 |  |
| --- | --- | --- | --- | --- |
|  | Very Difficult | 3 | 10.3 |  |
|  | Difficult | 6 | 20.7 |  |
|  | Somewhat Easy | 9 | 31.0 |  |
|  | Easy | 6 | 20.7 |  |
|  | Very Easy | 4 | 13.8 |  |
|  | Extremely Easy | 1 | 3.4 |  |
| **How easy or difficult is it to plan when you will use the medication each time?** | Extremely Difficult | 0 | 0.0 |  |
|  | Very Difficult | 1 | 3.4 |  |
|  | Difficult | 5 | 17.2 |  |
|  | Somewhat Easy | 9 | 31.0 |  |
|  | Easy | 6 | 20.7 |  |
|  | Very Easy | 5 | 17.2 |  |
|  | Extremely Easy | 3 | 10.3 |  |
| **How convenient or inconvenient is it to take the medication as instructed?** | Extremely Inconvenient | 1 | 3.4 |  |
|  | Very Inconvenient | 2 | 6.9 |  |
|  | Inconvenient | 11 | 37.9 |  |
|  | Somewhat Convenient | 4 | 13.8 |  |
|  | Convenient | 6 | 20.7 |  |
|  | Very Convenient | 4 | 13.8 |  |
|  | Extremely Convenient | 1 | 3.4 |  |
| **Overall, how confident are you that taking this medication is a good thing for you?** | Not at All Confident | 2 | 6.9 |  |
|  | A Little Confident | 0 | 0.0 |  |
|  | Somewhat Confident | 7 | 24.1 |  |
|  | Very Confident | 10 | 34.5 |  |
|  | Extremely Confident | 10 | 34.5 |  |

| **How certain are you that the good things about your medication outweigh the bad things?** | Not at All Certain | 2 | 6.9 |  |
| --- | --- | --- | --- | --- |
|  | A Little Certain | 1 | 3.4 |  |
|  | Somewhat Certain | 5 | 17.2 |  |
|  | Very Certain | 9 | 31.0 |  |
|  | Extremely Certain | 12 | 41.4 |  |
| **Taking all things into account, how satisfied or dissatisfied are you with this medication?** | Extremely Dissatisfied | 0 | 0.0 |  |
|  | Very Dissatisfied | 1 | 3.4 |  |
|  | Dissatisfied | 2 | 6.9 |  |
|  | Somewhat Satisfied | 6 | 20.7 |  |
|  | Satisfied | 5 | 17.2 |  |
|  | Very Satisfied | 5 | 17.2 |  |
|  | Extremely Satisfied | 10 | 34.5 |  |
| **TSQM Domain Scores** |  |  |  |  |
| **Side Effects (0-100)** | Mean, SD | 89.0 | 20.0 |  |
|  | Median, IQR | 100 | 12.5 |  |
|  | Minimum, maximum | 18.8 | 100 |  |
| **Effectiveness (0-100)** | Mean, SD | 70.5 | 21.3 |  |
|  | Median, IQR | 72.2 | 27.8 |  |
|  | Minimum, maximum | 16.7 | 100 |  |
| **Convenience (0-100)** | Mean (SD) | 54.2 | 20.2 |  |
|  | Median (IQR) | 55.6 | 27.8 |  |
|  | Minimum, maximum | 22.2 | 100 |  |
| **Global Satisfaction (0-100)** | Mean, SD | 73.4 | 25.8 |  |
|  | Median, IQR | 78.6 | 42.9 |  |
|  | Minimum, maximum | 7.1 | 100 |  |

*Only administered to subjects who reported experiencing side effects as the result of their medication (n = 13).

df, degrees of freedom; IQR, interquartile range; SD, standard deviation; TSQM, Treatment Satisfaction with Medications Questionnaire.

**Supplemental Table 6. Concomitant Medication Usage**

| **Medication Use (excluding asfotase alfa)** | **Baseline** | | **Month 3** | | **Month 6** | | **Month 12** | |  |
| --- | --- | --- | --- | --- | --- | --- | --- | --- | --- |
|  | **n = 50** | | **n = 49** | | **n = 40** | | **n = 29** | |  |
| **All Use, n (%)** |  |  |  |  |  |  |  |  |  |
| **Analgesics** | 10 | 20.0 | 9 | 18.4 | 7 | 16.3 | 3 | 10.3 |  |
|  |  |  |  |  |  |  |  |  |  |
|  |  |  |  |  |  |  |  |  |  |
|  |  |  |  |  |  |  |  |  |  |
| **Anti-inflammatory agents (excluding Flonase and Montelukast)** | 18 | 36.0 | 17 | 34.7 | 16 | 37.2 | 9 | 31.0 |  |
|  |  |  |  |  |  |  |  |  |  |
|  |  |  |  |  |  |  |  |  |  |
|  |  |  |  |  |  |  |  |  |  |
| **Anticonvulsant** | 6 | 12.0 | 5 | 10.2 | 5 | 11.6 | 1 | 3.4 |  |
|  |  |  |  |  |  |  |  |  |  |
|  |  |  |  |  |  |  |  |  |  |
|  |  |  |  |  |  |  |  |  |  |
| **Antidepressants** | 16 | 32.0 | 14 | 28.6 | 12 | 27.9 | 8 | 27.6 |  |
|  |  |  |  |  |  |  |  |  |  |
|  |  |  |  |  |  |  |  |  |  |
|  |  |  |  |  |  |  |  |  |  |
| **Muscle relaxants (skeletal)** | 3 | 6.0 | 3 | 6.1 | 2 | 4.7 | 2 | 6.9 |  |
|  |  |  |  |  |  |  |  |  |  |
|  |  |  |  |  |  |  |  |  |  |
|  |  |  |  |  |  |  |  |  |  |
| **PRN Use,**^a^ **n (%)** |  |  |  |  |  |  |  |  |  |
| **Analgesics** | 2 | 20.0 | 2 | 22.2 | 1 | 14.3 | 1 | 33.3 |  |
|  |  |  |  |  |  |  |  |  |  |
|  |  |  |  |  |  |  |  |  |  |
|  |  |  |  |  |  |  |  |  |  |

| **Anti-inflammatory agents (excluding Flonase and Montelukast)** | 3 | 16.7 | 2 | 11.8 | 3 | 18.8 | 2 | 22.2 |  |
| --- | --- | --- | --- | --- | --- | --- | --- | --- | --- |
|  |  |  |  |  |  |  |  |  |  |
|  |  |  |  |  |  |  |  |  |  |
|  |  |  |  |  |  |  |  |  |  |
| **Anticonvulsant** | 1 | 16.7 | 1 | 20.0 | 1 | 20.0 | 0 | 0.0 |  |
|  |  |  |  |  |  |  |  |  |  |
|  |  |  |  |  |  |  |  |  |  |
|  |  |  |  |  |  |  |  |  |  |
| **Antidepressants** | 0 | 0.0 | 0 | 0.0 | 0 | 0.0 | 0 | 0.0 |  |
|  |  |  |  |  |  |  |  |  |  |
|  |  |  |  |  |  |  |  |  |  |
|  |  |  |  |  |  |  |  |  |  |
| **Muscle relaxants (skeletal)** | 0 | 0.0 | 0 | 0.0 | 0 | 0.0 | 0 | 0.0 |  |
|  |  |  |  |  |  |  |  |  |  |
|  |  |  |  |  |  |  |  |  |  |
|  |  |  |  |  |  |  |  |  |  |
| **Acute Use**^a^ **(<30 days), n (%)** | |  |  |  |  |  |  |  |  |
| **Analgesics** | 2 | 20.0 | 1 | 11.1 | 0 | 0.0 | 0 | 0.0 |  |
|  |  |  |  |  |  |  |  |  |  |
|  |  |  |  |  |  |  |  |  |  |
|  |  |  |  |  |  |  |  |  |  |
| **Anti-inflammatory agents (excluding Flonase and Montelukast)** | 1 | 5.6 | 1 | 5.9 | 1 | 6.3 | 0 | 0.0 |  |
|  |  |  |  |  |  |  |  |  |  |
|  |  |  |  |  |  |  |  |  |  |
|  |  |  |  |  |  |  |  |  |  |
| **Anti-convulsant** | 0 | 0.0 | 0 | 0.0 | 0 | 0.0 | 0 | 0.0 |  |
|  |  |  |  |  |  |  |  |  |  |
|  |  |  |  |  |  |  |  |  |  |
|  |  |  |  |  |  |  |  |  |  |
| **Antidepressants** | 0 | 0.0 | 0 | 0.0 | 0 | 0.0 | 0 | 0.0 |  |
|  |  |  |  |  |  |  |  |  |  |
|  |  |  |  |  |  |  |  |  |  |
|  |  |  |  |  |  |  |  |  |  |
| **Muscle relaxants (skeletal)** | 0 | 0.0 | 0 | 0.0 | 0 | 0.0 | 0 | 0.0 |  |
|  |  |  |  |  |  |  |  |  |  |
|  |  |  |  |  |  |  |  |  |  |
|  |  |  |  |  |  |  |  |  |  |
| **Chronic Use**^a^ **(≥30 days) , n (%)** | |  |  |  |  |  |  |  |  |
| **Analgesics** | 6 | 60.0 | 6 | 66.7 | 6 | 85.7 | 2 | 66.7 |  |
|  |  |  |  |  |  |  |  |  |  |
|  |  |  |  |  |  |  |  |  |  |
|  |  |  |  |  |  |  |  |  |  |
| **Anti-inflammatory agents (excluding Flonase and montelukast)** | 14 | 77.8 | 14 | 82.4 | 12 | 75.0 | 7 | 77.8 |  |
|  |  |  |  |  |  |  |  |  |  |
|  |  |  |  |  |  |  |  |  |  |
|  |  |  |  |  |  |  |  |  |  |
| **Anticonvulsant** | 5 | 83.3 | 4 | 80.0 | 4 | 80.0 | 1 | 100 |  |
|  |  |  |  |  |  |  |  |  |  |
|  |  |  |  |  |  |  |  |  |  |
|  |  |  |  |  |  |  |  |  |  |
| **Antidepressants** | 16 | 100 | 14 | 100 | 12 | 100 | 8 | 100 |  |
|  |  |  |  |  |  |  |  |  |  |
|  |  |  |  |  |  |  |  |  |  |
|  |  |  |  |  |  |  |  |  |  |
| **Muscle relaxants (skeletal)** | 3 | 100 | 3 | 100 | 2 | 100 | 2 | 100 |  |
|  |  |  |  |  |  |  |  |  |  |
|  |  |  |  |  |  |  |  |  |  |
|  |  |  |  |  |  |  |  |  |  |

^a^Results reflect the percentage of medications being taken (either acutely, chronically, or PRN) among all patients with valid data.

**Supplemental Table 7. Asfotase alfa discontinuation**

|  |  | **Month 3** | | **Month 6** | | **Month 12** | |
| --- | --- | --- | --- | --- | --- | --- | --- |
|  |  | **n = 50** | | **N = 48** | | **n = 37** | |
| **Stopped taking asfotase alfa?** | Yes | 2 | 4.0 | 4 | 8.3 | 4 | 10.8 |
|  | No | 48 | 96.0 | 37 | 77.1 | 28 | 75.7 |
|  | Missing | 0 | 0.0 | 7 | 14.6 | 5 | 13.5 |
| **Why stopped taking asfotase alfa?** | Cost of prescription | 0 | 0.0 | 0 | 0.0 | 0 | 0.0 |
|  | Side effects | 2 | 100 | 1 | 25.0 | 0 | 0.0 |
|  | Lack of efficacy | 0 | 0.0 | 0 | 0.0 | 0 | 0.0 |
|  | Physician-recommended decision | 0 | 0.0 | 2 | 50.0 | 0 | 0.0 |
|  | Other | 0 | 0.0 | 1 | 25.0 | 3 | 75.0 |
|  | Unknown | 0 | 0.0 | 0 | 0.0 | 1 | 25.0 |
| Other at Month 6: "Did not like injection site side effects and fatigue."  Other at Month 12: "patient did not notice any difference on versus off the medication," "patient said Strensiq® did not seem to be working," and "patient's job switched insurance and stopped covering Strensiq." | | | | | | | |
